# Ethical and economic implications of the adoption of novel plant-based beef substitutes in the USA: a general equilibrium modelling study

**DOI:** 10.1016/S2542-5196(22)00169-3

**Published:** 2022-08-03

**Authors:** Daniel Mason-D'Croz, Anne Barnhill, Justin Bernstein, Jessica Bogard, Gabriel Dennis, Peter Dixon, Jessica Fanzo, Mario Herrero, Rebecca McLaren, Jeda Palmer, Travis Rieder, Maureen Rimmer, Ruth Faden

**Affiliations:** a Department of Global Development, College of Agriculture and Life Sciences, Cornell University, Ithaca, NY, USA; b Agricultural Economics and Rural Policy Group, Wageningen University & Research, Wageningen, Netherlands; c Berman Institute of Bioethics, Johns Hopkins University, Baltimore, MD, USA; d Vrije Universiteit Amsterdam, Amsterdam, Netherlands; e Commonwealth Scientific and Industrial Research Organisation, St Lucia, QLD, Australia; f Victoria University, Melbourne, VIC, Australia; g School of Advanced International Studies, Johns Hopkins University, Washington, DC, USA

## Abstract

**Background:**

Slowing climate change is crucial to the future wellbeing of human societies and the greater environment. Current beef production systems in the USA are a major source of negative environmental impacts and raise various animal welfare concerns. Nevertheless, beef production provides a food source high in protein and many nutrients as well as providing employment and income to millions of people. Cattle farming also contributes to individual and community identities and regional food cultures. Novel plant-based meat alternatives have been promoted as technologies that could transform the food system by reducing negative environmental, animal welfare, and health effects of meat production and consumption. Recent studies have conducted static analyses of shifts in diets globally and in the USA, but have not considered how the whole food system would respond to these changes, nor the ethical implications of these responses. We aimed to better explore these dynamics within the US food system and contribute a multiple perspective ethical assessment of plant-based alternatives to beef.

**Methods:**

In this national modelling analysis, we explored multiple ethical perspectives and the implications of the adoption of plant-based alternatives to beef in the USA. We developed USAGE-Food, a modified version of USAGE (a detailed computable general equilibrium model of the US economy), by improving the representation of sector interactions and dependencies, and consumer behaviour to better reflect resource use across the food system and the substitutability of foods within households. We further extended USAGE, by linking estimates of the environmental footprint of US agriculture, to estimate how changes across the agriculture sector could alter the environmental impact of primary food production across the whole sector, not only the beef sector. Using USAGE-Food, we simulated four beef replacement scenarios against a baseline of current beef demand in the USA: BEEF10, in which beef expenditure is replaced by other foods and three scenarios wherein 10%, 30%, or 60% of beef expenditure is replaced by plant-based alternatives.

**Findings:**

The adoption of plant-based beef alternatives is likely to reduce the carbon footprint of US food production by 2·5–13·5%, by reducing the number of animals needed for beef production by 2–12 million. Impacts on other dimensions are more ambiguous, as the agricultural workforce and natural resources, such as water and cropland, are reallocated across the food system. The shifting allocation of resources should lead to a more efficient food system, but could facilitate the expansion of other animal value chains (eg, pork and poultry) and increased exports of agricultural products. In aggregate, these changes across the food system would have a small, potentially positive, impact on national gross domestic product. However, they would lead to substantial disruptions within the agricultural economy, with the cattle and beef processing sectors decreasing by 7–45%, challenging the livelihoods of the more than 1·5 million people currently employed in beef value chains (primary production and animal processing) in the USA.

**Interpretation:**

Economic modelling suggests that the adoption of plant-based beef alternatives can contribute to reducing greenhouse gas emissions from the food system. Relocation of resources across the food system, simulated by our dynamic modelling approach, might mitigate gains across other environmental dimensions (ie, water or chemical use) and might facilitate the growth of other animal value chains. Although economic consequences at the country level are small, there would be concentrated losses within the beef value chain. Reduced carbon footprint and increased resource use efficiency of the food system are reasons for policy makers to encourage the continued development of these technologies. Despite this positive outcome, policy makers should recognise the ethical assessment of these transitions will be complex, and should remain vigilant to negative outcomes and be prepared to target policies to minimise the worst effects.

**Funding:**

The Stavros Niarchos Foundation, the Bill & Melinda Gates Foundation, Johns Hopkins University, the Commonwealth Scientific and Industrial Research Organisation, Cornell University, and Victoria University.

**Research in context**

**Evidence before this study**

No formal literature review was done. Global analyses and high-level reports have suggested that reductions in beef consumption, particularly in high-income countries (such as the USA), could contribute to reducing the environmental pressures of food production and consumption. Furthermore, a range of nationally focused studies have presented hypothetical scenarios of a shift away from beef in the USA and have suggested that dietary changes have substantial potential to reduce the environmental footprint of US food consumption. Novel plant-based alternatives have been marketed as a technological solution that could facilitate this transition, with lifecycle assessment studies suggesting notable environmental gains. However, these analyses have primarily applied static partial analyses that did not fully assess how agricultural producers could increase exports, and how resources freed from a decreasing beef sector might otherwise be used—both of which would have consequences on the environmental footprint of US food production. Furthermore, these studies have mostly focused on environmental and public health dimensions, with less focus on the ethical implications and the disruptive potential of these novel technologies, which might have unexpected consequences on value chains (eg resource allocation and employment) beyond just the beef sector.

**Added value of this study**

We build on previous static and partial analyses by way of a dynamic approach using a computable general equilibrium model of the entire US economy to simulate the interconnected nature of factor markets, economic activities, and economic outputs. Given the uncertainty in the potential adoption of plant-based alternatives, we explored various potential replacement scenarios, ranging from 10% replacement to 60%. We showed that plant-based beef alternatives should reduce the carbon footprint of the US food system, but that reductions across other environmental dimensions, such as water and cropland, will be dependent on how resources freed up from beef sectors are repurposed for other economic activities. These changes in resource availability would lead to disruptions across the US food system that negatively impact on employment for the many individuals working in beef sectors, as well as facilitating the expansion of other sectors.

**Implications of all the available evidence**

The threat of climate change is increasing, and the magnitude of the challenge to avoid 2°C of warming will require decarbonisation across all economic activities. Plant-based beef alternatives show substantial potential to reduce the carbon footprint of food systems where they are adopted. These novel technologies, if adopted widely, would negatively impact beef sectors with concentrated losses experienced by individuals and communities dependent on these economic activities. Nevertheless, these losses could be offset in aggregate from the expansion of other sectors (eg poultry, pork, and biofuels) in the food system. These changes would have ethically important implications, which policy makers should monitor to avoid severe unintended negative consequences to vulnerable workers, small producers, and hard-hit communities.

## Introduction

The food system has developed to supply more food to more people and at lower prices than at any point in recorded history. However, the unparalleled expansion of agricultural production has come at a cost, with substantial pressure on the environment through degradation of ecosystems and finite natural resources. The global food system is estimated to contribute 21–37% of global emissions,^1^, ^2^ with cattle accounting for an estimated 30–35% of global agricultural emissions, and with expansion of beef production identified as a key driver of land-use change and non-carbon dioxide (CO_2_) agricultural emissions. Attempts to avoid warming of about 1·5°C by the end of the century will require emissions reductions across the food system.^1^ In the USA, agriculture contributes about 10% of emissions,^3^ with direct emissions from cattle production accounting for about 40% of agricultural emissions^4^ or around 150 million tonnes (Mt) of CO_2_ equivalent (CO_2_eq) emissions. The Rockefeller Foundation estimated the US food system contributes about US$2·1 billion per year in externalised costs, with environmental and biodiversity costs accounting for more than $0·8 billion per year—much of which is driven by cattle emissions and land-use change.^5^

Beef production systems produce foods high in protein and many key nutrients, but which are over-consumed by many in the USA.^4^ These systems play an important sociocultural role, contributing to identity formation and regional food cultures; economically, they provide employment and income to millions, even as beef production raises various animal welfare concerns.^6^

Beef production and consumption have been the target of various real and hypothetical policies and food system interventions to reduce the environmental and public health impacts of the food system.^4^, ^7^, ^8^, ^9^ Novel plant-based alternatives are potentially transformative innovations that could reduce the environmental footprint of diets, by replacing animal products with less resource-intensive alternatives, and substantially reduce the number of animals raised for food. Reducing animal production and consumption could also reduce some negative public health risks (eg, risk of cardiovascular disease, spread of zoonoses, and foodborne pathogens).^10^, ^11^ Multiple plant-based alternatives from companies such as Meatless Farm, Very Good Food, and Impossible Burgers are produced using sophisticated processing technologies to mimic the taste, texture, and appearance of meat,^12^ and are increasingly available to consumers in many countries.^13^ Although these plant-based alternatives are currently more expensive than conventionally produced beef, the aforementioned companies have been reducing their prices and aim to reach price parity with conventional beef.^14^ According to one projection, by 2040, 25% of the global meat market could be plant-based alternatives.^12^

Transformative innovations can have positive and negative effects, which deserve ethical assessment. Critics of the burgeoning alternative protein industry, which includes plant-based alternatives and cellular agriculture, argue that it builds on a corporate food system that is unsustainable and inequitable.^10^, ^15^ Others see these as ethically promising innovations, which might help to solve environmental and animal welfare challenges, but with potential ethical trade-offs that must be considered.^16^, ^17^ These trade-offs could include negative effects on the producers of animal-sourced foods, and regional economies and communities based around animal-sourced food production. From an ethical perspective, it is crucial that evidence and approaches are developed to assess these trade-offs.

Here, we aimed to demonstrate an approach to assess some of the potential consequences of replacing beef demand in the USA with novel plant-based alternatives that attempt to mimic the taste and texture of beef. Many studies at the global^18^, ^19^, ^20^, ^21^ and national^22^, ^23^, ^24^, ^25^, ^26^ level have assessed the potential environmental and health benefits of dietary changes. However, most of these studies, particularly at the national level,^22^, ^23^, ^24^, ^25^, ^26^ have applied static and partial analyses and have not attempted to simulate how changes in consumer behaviour would drive a wide range of changes across the food system, or the potential ethical implications of these changes. The present study takes a dynamic and multidisciplinary approach, applying a computable general equilibrium model to simulate and assess how the food system and economy might adapt to reduced demand for beef and increased demand for plant-based alternatives, and contextualising these findings within multiple ethical perspectives.

Four scenarios are explored and are presented as deviations from a baseline scenario simulating current beef demand in the USA: BEEF10, in which 10% of beef expenditure is replaced by other foods, and three scenarios wherein 10%, 30%, or 60% of beef expenditure is replaced by plant-based alternatives. This range of beef reduction spans previous studies that have taxed food based on emissions^27^ and health outcomes,^4^ and projections on the potential market share of meat alternatives by mid-century (2040–50).^12^

## Methods

### Analytical framework

**Figure 1:**
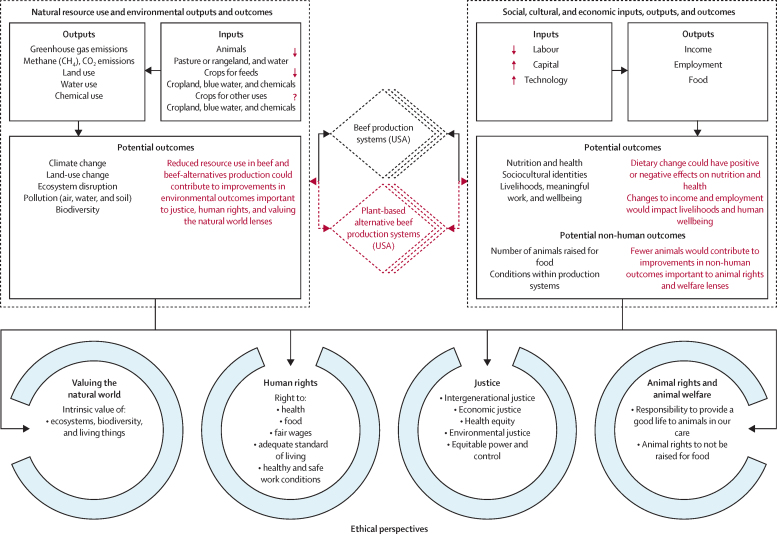
Analytical framework for a multi-perspective assessment of selected environmental and social outcomes of replacing beef with plant-based alternatives in the USA Here, we conceive the beef production systems as provisioning systems that connect resource use and outcomes. Each box highlights key causal chains linking inputs with ethically relevant outcomes, along with hypothesised changes in input use that can be tested (red arrows and ? icon) and potential changes in outcomes (red text) that can be assessed across ethical perspectives (blue rings).

In our analytical framework to inform a multi-perspective ethical assessment of the adoption of plant-based alternatives in the USA, beef and plant-based alternative production systems are conceptualised as provisioning systems with a series of inputs, outputs, and outcomes, which matter across multiple ethical perspectives. This framework includes impact pathways—ie, causal chains linking biophysical, social, cultural, and economic resource use and outcomes—which can be systematically explored via models (figure 1).

Previous ex ante assessments of plant-based beef alternatives using static projections (ie, without dynamic sector adjustments) have suggested wider use of these technologies, versus current use, would result in reductions in natural resource use across multiple dimensions and in the numbers of animals needed for food production.

Adoption of plant-based alternatives would disrupt value chains (thus changing relationships between food production and natural resource use) and the type and quantity of agricultural workforce (labour) and capital, leading to economic dislocations of ethical relevance. The outcomes of these changes would be relevant to humans, non-human animals, and the environment, and can be evaluated from multiple ethical perspectives. For example, changes in nutrition and health, livelihoods, and safe work all matter from multiple perspectives, including human rights, as they bear on recognised rights to health, food, an adequate standard of living, fair wages, and safe and healthy working conditions.^28^ From the perspective of justice (eg, health equity and economic justice), the consequences of changes in the food system are central to human wellbeing and equitable outcomes.^29^ Environmental outcomes are also relevant from multiple ethical perspectives. In addition to such outcomes being notable on the basis of the intrinsic value of the natural world (ie, ecosystems, biodiversity, and individual living things),^30^, ^31^ climate change can violate the rights of humans and contribute to intergenerational injustice.^32^ Outcomes for non-human animals, such as the conditions in which they are raised, matter from the perspective of animal rights and animal welfare.^33^, ^34^ Appendix 1 (p 1) includes a definition of animal welfare.

This use of multiple ethical perspectives is key to rigorous ethical evaluation. Stakeholders, advocates, and experts might bring different ethical perspectives to the evaluation of new technologies; some groups might reject the ethical perspectives embraced by others. Given this plurality of moral values, which is found within individual societies and across the global food system, the strongest ethical case for adopting a technology is not based on a particular value (eg, health equity) or one that assumes the authority of one ethical perspective (eg, human rights). Rather, the most robust ethical case is one that considers multiple, distinct ethical perspectives.

Figure 1 highlights such a multi-perspective approach to test the hypothesised benefits of plant-based alternatives and to explore some of the potential social and economic outcomes from the adoption of these technologies across various ethical perspectives.

### Scenario specification

Projecting the future of alternative plant-based proteins is complex. Challenges include substantial uncertainty about how quickly and to what extent these technologies will be adopted, and for how long they might remain relevant. Even defining these technologies is difficult, as this novel sector has a demonstrated ability to quickly adapt production practices in response to consumer concerns around taste and nutritional content.^35^, ^36^

To better address this uncertainty, we used four scenarios to explore the possibility space around the adoption of novel alternative meat products in the USA. We simulated three scenarios in which consumers replace current beef demand with plant-based alternatives at three different levels: 10% (ALTP10), 30% (ALTP30), and 60% (ALTP60) replacement. As future plant-based technologies might differ from current iterations, we will represent the adoption of these novel products as increasing demand for the plant-based constituents (grains, legumes, oilseeds, and starches) listed as ingredients by current plant-based protein companies.^25^, ^37^ Appendix 1 (p 2) lists specific commodities that are included in the set of potential inputs to novel alternative meat products. Additionally, we simulated a scenario wherein 10% of beef demand is replaced with other foods in a household's current consumption bundle (BEEF10). Given the role of the USA as a major agricultural exporter, we conducted sensitivity testing of varying levels of agricultural trade. Full details are given in appendix 1 (p 30).

This replacement range of 10–60% provides a plausibility space of development of the plant-based meat market that is currently small in comparison to the overall meat market (1·4% of meat category) but is a rapidly growing market.^38^ The range is bounded on the high end by industry-based projections that meat alternatives could achieve market shares of up to 60% by 2040.^12^ The 10% scenario was selected to facilitate future comparison with sustainable diet literature, which have targeted reductions in beef and red meat consumption using emission-based and health-based taxation.^4^, ^27^

All four beef-demand scenarios are deviations from a baseline scenario, which simulates a business-as-usual scenario of the current economic state of the food system in the USA. A detailed description of the USAGE-Food model is given in appendix 1 (p 6). Changes in beef demand are the only deviation from baseline assumptions, such that all changes in model outcomes can be attributed to the change in consumer demand. In all four beef-demand scenarios, we model the change in beef demand by imposing an exogenous preference shift so that we can see how the food system would respond to a specific substitution of beef with either plant-based alternatives (ALTP10–ALTP60) or other food commodities. This substitution is applied in a symmetrical shift to ensure there is no exogenous preference shift between food and non-food expenditure.

### Economic modelling

Although agriculture accounts for less than 2% of total US employment, the broader food system (including food manufacturing) employs 11%,^39^ and accounts for more than 50% of land use and 80% of consumptive water use.^40^ We aimed to understand how the introduction of novel plant-based alternatives at scale could disrupt supply chains, shifting resource availability and allocation across the economy. To do so, we modified the USAGE model,^41^, ^42^, ^43^ a computable general equilibrium model of the US economy, to develop a version of the model (USAGE-Food) with more detailed countrywide representation of the US food system. The USAGE-Food model was run using GEMPACK 12.1. Using a detailed computable general equilibrium model, such as USAGE-Food, has the benefit of detailed sector representation—for example, we ran the model for 392 industries and commodities, 27 of which are food-related—which we can then aggregate to highlight how these sectors relate to the overall economy. However, focusing on expanding the sector representation required running the model without detailed regional disaggregation in this instance. Commodities and aggregations are listed in appendix 1 (p 21).

USAGE-Food includes two key improvements to USAGE that facilitate analysis of the US food system. First, on the production side, we disaggregated the beef value chain from other animal meat value chains (eg, pork and lamb), thus creating an appropriate industry nesting in production functions to capture economic ties between various meat sectors and the rest of the economy. This nesting allowed us to better represent how changes in beef production would spill over to the rest of the food system. A stylised representation of nesting is given in appendix 1 (p 4). Second, we updated and improved the representation of food demand by specifying in greater detail the substitutability of different types of foods in the household utility function, adding more detailed nesting between food and non-food commodities, and between various meat (ie, beef, poultry, and pork) and non-meat commodities within the food nest. To quantify these changes to the utility function we updated the constant elasticity of substitution functions to reflect a range of food demand studies of the USA^44^ and global meta-analyses of food demand.^45^, ^46^

### Estimating environmental impacts

To estimate changes along environmental dimensions, we constructed a baseline environmental footprint for agricultural production in the USA. First, we used agricultural production data from FAOSTAT,^47^ which was consistent with both USAGE and the environmental coefficients of production used in the EAT–*Lancet* Commission on healthy diets from sustainable food systems.^19^, ^20^

We aggregated the environmental footprint of agricultural production in the USA to USAGE-Food agricultural commodities (table 1). Once this baseline was established, we estimated changes along environmental dimensions based on the changes in production simulated in USAGE-Food. Detailed commodity mapping between Springmann and colleagues^19^ and USAGE-Food is presented in appendix 1 (p 3).

**Table 1:** Baseline agricultural resource use in the USA, by USAGE-Food agricultural commodity

		**Blue water use (km^3^)**	**N application (000 tonnes)**	**P application (000 tonnes)**	**Greenhouse gases (Mt CO_2_ eq)**
Animal products					
	Live cattle	..	..	..	142
	Dairy cattle	..	..	..	46
	Live poultry	..	..	..	25
	Other live animals	..	..	..	30
Crops					
	Grain farms	46	8320	1244	69
	Fruit and nut farms	7	103	13	1
	Vegetable or melon farms	9	229	62	6
	Oilseed farms	18	205	180	13
	Other crops	14	1210	137	3
Food processing					
	Soya oil processing	0	0	0	10
	Sugar processing	0	0	0	2
Total		94	10 067	1636	347

Environmental coefficients from the EAT–*Lancet* Commission^19^, ^20^ were used to estimate the direct resource use of food production. Where values are blank (eg, blue water use for animal products), it is because blue water was accounted for in the irrigation of feed crop as opposed to a direct input to animal production. CO_2_ eq=carbon dioxide equivalent. Mt=million tonnes.

### Role of the funding source

The funders of the study had no role in study design, data collection, data analysis, data interpretation, or writing of the report.

## Results

**Figure 2:**
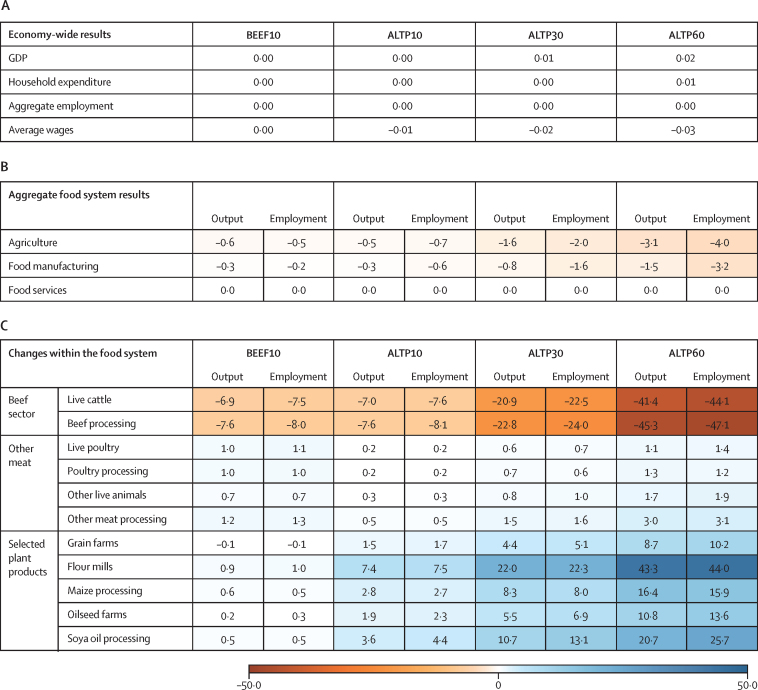
Summary of changes in key economic variables by scenario and sector Results are reported as percent change from the baseline scenario simulating current beef demand. Household expenditure refers to the value of total household expenditure on final goods, but excludes savings and government spending. Output refers to the value of production. Employment refers to aggregate employment and reflects changes in employment rate across the entire economy. Sector employment reflects employment in the specified sector. Employment is expressed in USAGE in hours of agricultural workforce labour. Wages reflect the average wage across the whole of the economy. ALTP10=10% of beef expenditure substituted with plant-based alternatives. ALTP30=30% of beef expenditure substituted with plant-based alternatives. ALTP60=60% of beef expenditure substituted with plant-based alternatives. BEEF10=10% reduction in beef expenditure without any novel products. GDP=gross domestic product.

Changes in gross domestic product, household expenditure, aggregate employment, and wages were seen under all four scenarios (figure 2A). Across the food system, employment in and the overall value of production (output) declines across all of agriculture (0·5–4·0%) and food manufacturing (eg, food processing and packaging; 0·2–3·2%) sectors, with neutral impacts on food services (eg, restaurants; figure 2B). However, these aggregate impacts mask substantial disruption within the food system as it responds to changes in consumer food demand.

Within the food system, the beef-producing and processing sectors are most negatively affected by reductions in consumer demand for beef, with output declining by about two-thirds of the reduction in demand: a 7% reduction in the BEEF10 and ALTP10 scenarios, and a reduction of up to 40% in the ALTP60 scenario (figure 2C). This smaller decline in production is due to there being various uses for beef products besides human consumption (eg, pet food and cosmetics) and the role of the USA as a major exporter of beef and other agricultural goods. Our sensitivity analysis on trade assumptions can be found in appendix 1 (p 30).

Reductions in domestic demand for beef and beef products drives a reduction in sector output, which in turn drives down the demands by these sectors for inputs, such as agricultural workforce, capital, and animal feed. Reduced demand does not translate entirely to a reduction in output, mostly due to an increase in exports of 2·5–21·0% across the four scenarios, with the beef sector becoming increasingly export-oriented with 14–37% of output exported compared with around 12% in the baseline scenario. Full details can be found in appendix 1 (p 4). Across all scenarios, we saw a substantial reduction in employment in the beef value chains, which currently employ around 1 million people in cattle operations and 0·5 million in animal processing.^48^

As beef-producing sectors contract, agricultural inputs (ie, land, labour, and water) are freed up to be used in the production and provision of other commodities and services, with most of this reallocation occurring among other sectors in the food system. Under the alternative protein scenarios (ALTP10–ALTP60) much of these resources are reallocated to alternative protein value chains. Relatively large increases are seen in output and employment in grain and oilseed processing sectors, which would supply key inputs to the production of plant-based products.

Other animal sectors expand production in response to growing demand and, because the decreasing beef sector makes many key inputs more available, production costs are decreased. Due to shifts in production cost and consumer preferences, production increases in other animal sectors across all four scenarios. In BEEF10, the decrease in beef demand is accompanied by increased demand for other animal-sourced foods (such as poultry and pork). This increased demand leads to an expansion of around 1% for these sectors, with an associated increase in employment and increased demand for commercial feeds (such as grains and oilseeds). In the three ALTP scenarios, the expansion of other animal sectors (range 0·2–1·7%) is driven primarily through declining production costs. In the USA in 2019, more than 8·5 billion chickens and 70–80 million pigs were reared for meat production.^49^ The projected changes in production across the four scenarios could lead to an annual decline in cattle numbers of 2–12 million, with accompanied increases of 16–94 million chickens and 0·2–1·4 million pigs. Notably, at similar levels of beef replacement, replacing beef with other animal products (BEEF10 scenario) leads to 2–5 times more chickens and pigs than when replaced with plant-based alternatives (ALTP10 scenario).

**Figure 3:**
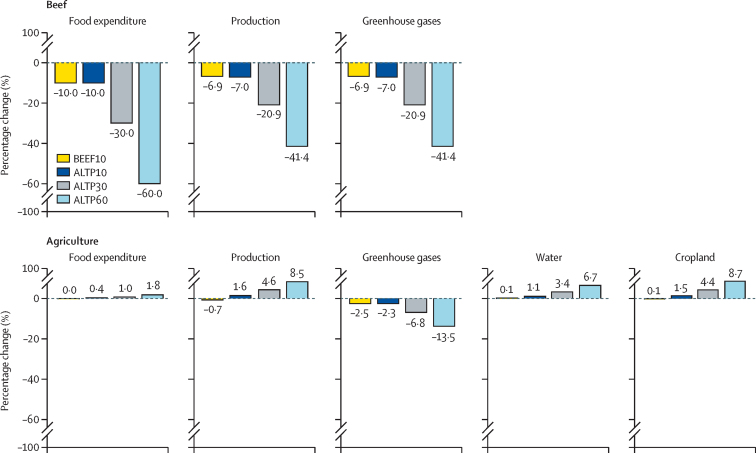
Percentage change across selected environmental dimensions from baseline in the beef sector and across all agricultural sectors under a range of scenarios Food expenditure is given in US$. Production is given in tonnes. Greenhouse gases are reported as annual emissions in CO_2_eq. Water represents changes in blue water use in km^3^. Cropland is reported in 1000 hectares of harvest area. Blue water and cropland are not used directly by livestock, but are used instead for crop production (some of which serves as feed). ALTP10=10% of beef expenditure substituted with plant-based alternatives. ALTP30=30% of beef expenditure substituted with plant-based alternatives. ALTP60=60% of beef expenditure substituted with plant-based alternatives. BEEF10=10% reduction in beef expenditure without any novel products. CO_2_eq=carbon dioxide equivalent.

Our results are consistent with suggestions that plant-based alternatives could reduce greenhouse gas emissions, with direct emissions from beef production declining by 7% to 41% (figure 3) or by 10 Mt or 61 Mt CO_2_eq emissions per year, respectively, for the ALT10 and ALT60 scenarios. However, these reductions are partially attenuated due to increased production from other sectors (figure 2), with overall agricultural emissions declining by 2·5–13·5% or 7–40 Mt CO_2_eq emissions per year. These results also suggest a potential shift in the composition of agricultural emissions, with conventional beef production in the USA estimated to have a carbon footprint of 16–40 kg CO_2_eq per kg of beef.^50^ This footprint is primarily composed of potent, but shorter-lived, methane and nitrous oxide, compared with estimates of the carbon footprint of novel plant-based alternatives (3–4 kg CO_2_eq per kg; mainly driven by energy usage).^25^, ^37^

The impacts across other environmental dimensions are more ambiguous than changes in emissions. Most of the water, cropland, and chemicals (ie, fertilisers and pesticides) associated with beef production are used in the production of feed crops (cereals and oilseeds primarily).^50^ If cereal and oilseed production do not decline, then natural resource use across various dimensions is unlikely to change dramatically. Our modelling (figure 3) suggested there could be a small increase in water (range 0–7%) and cropland use (range 0–9%) across the four scenarios. This projected increase was due to an expansion in other agricultural sectors, given that many commercial feed crops are important inputs not only to the beef sector but to other animal sectors, novel plant-based alternatives, and a range of non-food uses (eg, alcohol, biofuels, and starches). These crops are also part of complex global value chains with feed crops traded at higher rates than animal products.^51^ Projected changes in fertiliser application followed changes in water and cropland use (appendix 1 p 5). Change across total agricultural land use (cropland and pastureland) would probably decline under all four scenarios, due to the large land footprint of conventional beef production.

Notably, although total changes in agricultural resource use are small, shifting from conventional beef production to plant-based alternatives should increase the overall resource efficiency of the food system, as these products—and other animal products like poultry and pork—are more efficient in converting material inputs into protein and have a smaller environmental footprint. Many of these changes suggest ethically relevant trade-offs across the various ethical lenses highlighted in figure 1. Table 2 summarises some of the ethical implications of these modelling results.

**Table 2:** Summary of selected modelled results, key points of uncertainty, and ethical implications of the replacement of beef with plant-based alternatives

	**Modelled results of substituting beef with plant-based alternatives**	**Key points of uncertainty**	**Considering potential outcomes from multiple ethical perspectives**
**Planetary boundaries**			
Greenhouse gases	Overall reduction in CO_2_ eq driven by reduction in non-CO_2_ emissions	Will more energy-intensive food processing increase demand for electricity, with subsequent increase in CO_2_ emissions from electricity generation? Will wide adoption of plant-based alternatives to non-beef animal products have less of an impact on emissions reductions, given that conventionally produced pork and poultry have comparable carbon efficiency to plant-based alternatives?	(1) Shifts in production could lead to reductions in warming potential, and contribute to climate change mitigation (justice: intergenerational, economic, health equity, environmental; VNW; human rights: health, food, standard of living); (2) a more resource-efficient food system could sustain more people with a given resource level (justice; human rights: food); (3) changes in demand and production, reductions of total resource use by the food system could reduce the environmental impact of the food system across some environmental dimensions. (justice: intergenerational; VNW; human rights: health, food, standard of living, healthy and safe working conditions)
Use of agricultural inputs (land, water, chemicals)	Small changes in cropland, water, and chemical use	Will overall resources use decline after resource allocation to other uses? Reduction in pastureland likely unless other uses arise	*
**Animal welfare**			
Animal numbers	Fewer number of beef cattle; increased numbers of other animals (eg, pigs and chickens)	Will plant-based beef alternatives compete with other beef (and meat) alternatives? Will plant-based beef alternatives compete with other meats? Will the wide adoption of other plant-based alternatives more significantly reduce animal numbers?	(1) Plant-based beef alternatives could lead to more chickens and pigs raised for food, who are more often raised in confinement conditions (ARW); (2) the adoption of other plant-based alternatives could more significantly reduce animal numbers (ARW)
**Economy and livelihoods**			
GDP	Neutral impact on aggregate GDP; growing sectors offset declining sectors	What constraints could prevent reallocation of resources between sectors? What would be the consequences of the more disruptive adoption of a wider array of plant-base alternatives?	(1) The adoption of plant-based alternatives to beef (and more broadly to other animal products) could contribute to changes in the access to resources and jobs across the economy (human rights**:** livelihoods); (2) the consequences of these changes would be heterogeneous, with some regions or individuals benefiting while others suffer from the changes, contributing to diverging economic, social, and health outcomes (human rights: standard of living; justice: economic)
Regional economic effects	Not assessed directly	What would be the regional consequences of disruptions in the food system? Where will plant-based alternatives ultimately be produced? Will there be novel sectors that could replace the economic role of animal production?	*
Animal-sourced food producers	The beef sector would contract substantially; reductions in producer prices could reduce profitability of the sector	How would beef producers adapt to a contraction in the beef sector (eg, through diversification or new income generating activities)? Would the adoption of other plant-based alternatives reduce options for income diversification? What will be the effects on agricultural producers' livelihoods, participation in meaningful work, and identities?	(1) If animal-sourced food producers cannot successfully adapt to a contraction in their sectors, they might cease operations altogether, leading to negative effects on their livelihoods, participation in meaningful work, and identities (human rights**:** livelihoods); (2) increased concentration of agricultural sectors could increase power asymmetries, and disproportionately affect smaller producers (justice: economic, equitable power and control)
Agricultural labour	Wages are steady across the scenarios; labour shifts from contracting sectors to expanding sectors	Are there constraints that limit labour mobility (between regions or sectors) and contribute to regional unemployment? What would be the work conditions for workers moving to new jobs? Will already marginalised workers (eg, migrant workers) be disproportionately negatively affected? The wider adoption of plant-based alternatives could require greater reskilling for labour to move to other jobs	(1) If labour mobility is constrained, workers in animal sectors could see negative impacts on their bargaining power, future wages, and ability to demand safer working conditions (human rights**:** standard of living, fair wages, working conditions), with marginalised groups more likely to be disproportionately affected (justice**:** economic); (2) diversification of economic activities could see shifts towards safer and more economically lucrative activities (human rights**:** working conditions, wages, livelihoods, health; justice: economic, health equity)
**Public health**			
Nutrition	Not assessed directly	What is the contribution of plant-based beef, and more broadly plant-based alternatives, to nutrition? What are the long-term consequences of their consumption on dietary quality and diet-related illness risk? Would declining prices for beef lead to rebound effects on beef consumption?	(1) There could be important public health consequences (positive or negative, or both) of the wide consumption of plant-based alternatives; (2) access to plant-based alternatives might not be equally available, which could have disproportionate (positive or negative) impacts across society, particularly if there are any rebound effects (justice: health equity)
Food safety and public health effects of animal production	Not assessed directly	What will be the aggregate effect of shifts in animal production on public health risks (eg, pollution, zoonotic risk, and food-based pathogens)? What risks will these novel processes introduce into the food system (eg, failures in industrial food processing similar to the 2022 baby formula recall)?	(1) Changes in agricultural production (what is produced and where) could contribute to changes in public health, with local and national consequences (human rights: health; justice: health equity, economic, environmental); (2) changing composition of diets would alter the food-based risks to public health with impacts likely to affect the population heterogeneously (human rights: food, health; justice: health equity)

Ethical perspectives follow from figure 1 and are given in brackets after each potential outcome. CO_2_ eq=carbion dioxide equivalent. GDP=gross domestic product. VNM=valuing the natural word. ARW=animal rights and animal welfare.

* Same as above.

## Discussion

Our results suggest the adoption of plant-based beef alternatives would reduce the carbon footprint of US food production, which is an ethically important outcome. However, reductions across the entire food system along other environmental dimensions (eg, water, land, and chemical inputs) are more ambiguous. Although plant-based alternatives use less land and water per unit of production, our modelling results suggest that many of these freed resources would be reallocated to other uses across the food system. This change would allow the food system to produce more with the same inputs (increased efficiency), but does not assure that total resource use would decline.

Economically, our results suggest a large shift to plant-based alternatives would have a neutral effect on gross domestic product, with gains in expanding sectors roughly offsetting losses in decreasing sectors. However, despite the minor effect in the aggregate, the distribution of positive and negative economic effects across the economy is ethically important. Whether these changes would increase economic inequality and exacerbate existing vulnerabilities warrants further investigation. Who ultimately adopts these products might vary by demographic and economic characteristics, concentrating impacts of consumption to certain consumers.^52^ Those currently involved in beef value chains are likely to experience most of the economic losses and dislocations caused by the shift towards plant-based alternatives. Large producers are responsible for most meat production and profits, even as most farms in the USA are classified as small family-owned farms, with the median cattle operation failing to break even, requiring off-farm income to supplement farm income.^53^, ^54^ It is unknown to what extent small farmers would be able to adapt to reduced beef demand by further diversification of economic activities, or if these changes would make continued involvement in the beef value chain untenable.

The economic model used in this study assumes that those employed in agricultural sectors can find employment in other economic sectors, but there can be constraints to labour mobility, particularly in the short term. Our results suggest that, although there would be substantial declines in employment in beef processing, much of this workforce could move to other animal processing sectors (eg, pork and poultry) without substantial reskilling required. Nevertheless, it is important to recognise concerns about working conditions across animal processing sectors, with workers experiencing high rates of injury.^55^, ^56^ Furthermore, the current structure of US agricultural production^49^ suggests these changes could require those employed in beef sectors moving from major beef producing regions (the Grain Plains, central USA) to regions specialising in pork and poultry (southeast USA). These dislocations could have real costs that are likely to fall disproportionately on disadvantaged people and communities, and should be the focus of future research. For example, these changes could have serious implications for the rural economies reliant on cattle ranching and beef processing and put new pressure on receiving communities that might not have the infrastructure to absorb incoming populations. Moreover, processing sectors for all animal products are heavily reliant on migrant labour, a particularly disadvantaged and vulnerable group.

Our modelling results also suggest there could be unintended consequences for animal welfare of adopting plant-based beef alternatives. The freeing up of resources used in beef production could facilitate the expansion of the pork and poultry sectors, thus swapping a relatively small number of cattle (2–12 million per year) for a much larger number of chickens (16–94 million per year) and pigs (0·2–1·4 million per year). There are two concerns here. First, the sheer numbers of animals affected, across all relevant species, could increase. Second, the welfare conditions of most pigs and chicken in agricultural production are arguably worse than those of cattle, given that the pork and poultry sector more frequently use confined feeding operations. As such, replacing beef with plant-based beef alternatives is preferable to replacing beef with other animal products, from the perspective of animal welfare. In our analysis, directly substituting beef with other animal products (BEEF10 scenario) resulted in 2–5 times more pigs and chickens than replacing beef with plant-based alternatives (ALTP10 scenario).

It is also important to consider the disruptive potential of the full range of alternative meat products. Plant-based alternatives to chicken and pork are increasingly coming to market and, if widely adopted together with beef alternatives, could more drastically reduce the animals in food production. Wide adoption of a range of alternatives would be economically more disruptive than our four modelled scenarios, in which only beef is replaced. For example, greater economic dislocation would be expected, since people employed in the beef processing sector would not be able to find employment in other animal processing sectors, requiring greater reskilling of the agricultural workforce to move to new activities.

In summary, the threat of climate change is increasing and plant-based alternatives could help to reduce the carbon footprint of the food system, while also reducing the number of animals needed to meet growing global food demand. If widely adopted, plant-based alternatives could restructure the food system, redefining economic and material flows within many value chains, with some economic activities expanding while others decrease. New demand for crops might spur intensification of crops that were previously less economically important (eg, pulses as inputs to plant-based alternatives). All these changes will ultimately lead to both positive and negative effects along many dimensions, all with important ethical implications.

This study has highlighted the importance of using dynamic approaches in addition to static lifecycle assessment-based approaches when assessing the ethical implications of potentially transformative innovations. Nevertheless, the work has some limitations. First, the detailed sector representation of USAGE-Food precluded the models from being run with detailed regional disaggregation. Second, although computable general equilibrium models are powerful modelling tools, like all models, they are simplifications of reality and can struggle to represent an economy out of equilibrium and represent behaviour primarily from an economic lens. As such, future research should apply multi-model assessments to advance understanding of important ethical questions that address some of the limitations of this study, including the following four priorities.

First, exploring a wider range of potential adoption pathways of plant-based alternatives, including how these novel products could compete with conventional animal products and with other existing and future novel protein products (eg, cultured meats). This research should consider heterogeneous demand preferences across the USA,^52^ and the potential for rebound effects in consumer demand in response to price changes.

Second, extending beyond the USA to consider the potential impact of increased global demand of plant-based alternatives on agricultural producers in low-income and middle-income countries.

Third, assessing the public health impacts of shifting human diets towards plant-based alternatives, including the distribution and frequency of zoonoses and foodborne pathogens, and the burden of disease from chronic illnesses.

Finally, modelling in greater detail the regional, household, and firm-level impacts of shifts to plant-based alternatives to help to develop transition pathways that restrict negative economic effects on individuals currently involved in the beef value chain.

Although future research is needed to assess the ethical implications of plant-based alternatives more fully, the results of our multi-dimensional assessment across multiple ethical perspectives show that shifts from beef to plant-based alternatives have the potential to deliver a number of moral goods, but also reveal that a full accounting of the ethics of such a transition will be complex. Our findings suggest that plant-based alternatives could play an important role in helping to reduce the carbon footprint of the food system and have the additional benefit of increased resource use efficiency, with relatively small (albeit concentrated) negative economic impacts. These are good reasons for regulators and policy makers to encourage these technologies at this early stage of development and adoption, but only if they remain vigilant to unintended negative consequences and commit to mitigating those that are ethically concerning, including harms to disadvantaged workers and hard-hit local communities and small producers.

## Data sharing

The USAGE-Food model output used in this study is summarised in appendix 2. Mathematical descriptions of modified components of the USAGE model are included in appendix 1 (pp 6–29). Documentation of USAGE is available at https://www.copsmodels.com/usage.htm. Full model simulations have been made available in the form of zipped archives in a Mendeley Data Repository available at: http://doi.org/10.17632/dmhhfw2yzt.1. The USAGE-Food model was run using GEMPACK 12.1, which requires a licence. A model-specific and scenario-specific GEMPACK licence to reproduce the model results can be made available. Inquiries to access this limited licence as well as for more detailed training in the use of the USAGE model can be addressed to PD (Peter.Dixon@vu.edu.au) and MR (Maureen.Bleazby@vu.edu.au).

## Declaration of interests

We declare no competing interests.
