# Supplementary material for: Ethical and economic implications of the adoption of novel plant-based beef substitutes in the USA: a general equilibrium modelling study
Source: Lancet Planet Health. 2022 Aug 3;6(8):e658–69. doi: 10.1016/S2542-5196(22)00169-3 (PMC9364141; doi:10.1016/S2542-5196(22)00169-3)
Supplement: Supplementary appendix [file mmc1.pdf]

THE LANCET
Planetary Health

Supplementary appendix 1

This appendix formed part of the original submission and has been peer reviewed. We post it as supplied by the authors.

Supplement to: Mason-D'Croz D, Barnhill A, Bernstein J, et al. Ethical and economic implications of the adoption of novel plant-based beef substitutes in the USA: a general equilibrium modelling study. *Lancet Planet Health* 2022; **6:** e658–69.

Ethical implications of alternative plant-based beef substitutes in the USA: a general equilibrium modelling study

Supplementary Materials

Table of Contents

S1. Defining Animal Welfare 1

S2. Supplementary Figures and Tables 2

S3. Model Documentation 6

Computable General Equilibrium Models 6

General Description of USAGE 6

Updating Food demand in USAGE 8

Production Functions 9

Utility Function 14

S4. Sensitivity of modelling results to changes in export specification of the beef sector 30

References 32

Supplementary Figures and Tables

Figure S1 Stylized representation of nesting in USAGE-FOOD production function 4
Figure S2 Nitrogen and phosphorous application for U.S. Agriculture by scenario 5

Table S1 Modelled constituents of alternative plant-based meats 2
Table S2 Mapping EAT-Lacet commodity coefficients to USAGE commodities 3
Table S3 Summary of changes in beef food demand, output, exports, and the export share of output by scenario 4
Table S4 Food commodities within food sub-sets in USAGE-Food 8
Table S5 Production function with 5-level CES nests: general case 10
Table S6 Input-demand functions in percentage change form 10
Table S7 Nesting assumptions for an industry production function in USAGE-Food: 12
Table S8 Number of items in the production function nests in USAGE-Food 13
Table S9 4-Level Utility Function 15
Table S10 Nesting assumptions for consumer utility in USAGE-Food 16
Table S11 Number of items in the consumer nests in USAGE-Food 17
Table S12 USAGE commodities and industries with mapping to 45 and 9 sector aggregations 21
Table S13 Sensitivity of results to changes in the export specification for the beef sector: percentage effects of a 10 percent reduction in household demand for Beef processing products 31

S1. Defining Animal Welfare

The adoption of plant-based alternatives to meat will change the numbers of different animals who are raised in different kinds of production systems. What animal welfare is or how it should be defined, and what it means for a farm animal to have a good life, are contested issues. Given this it is important that we define what we mean by animal welfare.

Over decades of discussion among animal welfare experts, ethicists, those involved in animal agriculture, and others, three distinct components of animal welfare emerging as important: "(1) that animals should be spared negative affect (pain, fear, hunger, etc.) as much as possible, and by experiencing positive affect in the form of contentment and normal pleasures; (2) that animals should be able to lead reasonably natural lives by being able to perform important types of normal behaviour and by having some natural elements in their environment such as fresh air and the ability to socialize with other animals in normal ways; and (3) that animals should function well in the sense of good health, normal growth and development, and normal functioning of the body." [1]

When assessing production systems, there are trade-offs between these dimensions of animal welfare. For example, pasture-based production systems can expose animals to predation and severe weather, potentially increasing mortality, and some kinds of negative affect, but also afford animals more opportunity to engage in species-typical behaviours and to experience positive affect as a result. Confinement operations, where animals are densely packed indoors, protect animals from predators and severe weather, but subject animals to other sources of injury and disease, may not allow animals much opportunity to engage in natural behaviours, and may subject animals to significant stress. These trade-offs notwithstanding, many ethicists have concluded that animals have lower welfare on average in confinement animal operations, and that the level of welfare generally afforded animals in confinement operations in the United States is ethically unacceptable[2,3]. From this ethical perspective, an increase in the number of animals in confinement systems is ethically undesirable.

S2. Supplementary Figures and Tables

Table S1 Modelled constituents of alternative plant-based meats

USAGE CODE	DESCRIPTION
**OILSEEDFARM**	Farms growing oilseeds (e.g., soybean, coconut, etc.) that provide fat and protein content to alternative plant-based meats
**GRAINFARM**<br>**OTHCROPFARM**	Farms growing grains (e.g., wheat, rice, etc.) and other crops (e.g., pulses, potatoes, etc.) that can provide protein and starches
**FLOURMALMILL**<br>**WETCORNMILL**<br>**SOYOILPROC**<br>**FATSOILS**	Sectors that process primary agricultural commodities produced on farms into a range of products including flour, starch, vegetable oils which are inputs into alternative plant-based meats

Table S2 Mapping EAT-Lacet commodity coefficients to USAGE commodities

COMMODITY GROUP	USAGE COMMODITY	EAT-LANCET COMMODITY	DESCRIPTION
**ANIMAL**	CattRanchFarm	cbeef	Beef production
	DairCattProd	cmilk	Dairy Production
	OtherAnimal	clamb	Sheep, Goat, Lamb Production
		cpork	Pork production
	PoultryEgg	ceggs	Egg production
		cpoul	Poultry Production
**CROPS**	FruitNutFarm	cbana	Banana production
		csubf	Sub-tropical and tropical fruit production
		ctemf	Temperate fruit production
	GrainFarm	cbarl	Barley production
		cmaiz	Maize production
		cmill	Millet production
		cocer	Other cereal production
		crice	Rice production
		csorg	Sorghum Production
		cwhea	Wheat Production
	OilseedFarm	cgrnd	Groundnut (peanut) production
		crpsd	Rapeseed (canola) production
		csnfl	Sunflower production
		csoyb	Soybean production
		ctols	Other oilseed production
	OthCropFarm	ccafe	Coffee production
		ccott	Cotton production
		cothr	Other crop production
		csugb	Sugar beet production
		csugc	Sugar cane production
	VegMelonFarm	cbean	Bean production
		cchkp	Chickpea production
		ccowp	Cowpea production
		clent	Lentil production
		copul	Other pulse and legume production
		corat	Other root and tuber production
		cpota	Potato production
		cswpt	Sweet potato production
		cvege	Vegetable Production
**FOOD PROCESSING**	SoyoilProc	cgdol	Groundnut oil production
		crpol	Rapeseed oil production
		csbol	Soybean oil production
		csfol	Sunflower oil production
		ctool	Other vegetable oil production
	SugarConfec	csugr	Sugar production

Table S3 Summary of changes in beef food demand, output, exports, and the export share of output by scenario

	IMPOSED CHANGE IN BEEF FOOD DEMAND (%)	CHANGE IN BEEF OUTPUT (%)	CHANGE IN BEEF EXPORTS (%)	EXPORT SHARE OF OUTPUT
**BEEF10**	-10.0	-7.6	2.5	13.9
**ALTP10**	-10.0	-7.6	2.5	13.9
**ALTP30**	-30.0	-22.8	8.5	18.9
**ALTP60**	-60.0	-45.3	21.0	37.0

The change in output is about ⅔ the reduction in food demand, as a portion of excess output is exported. This increase in exports mitigates some of the decline in demand. However, it does not prevent the beef sector from contracting, even as it becomes increasingly export oriented as the share of output that is exported increases from 12.2% in the baseline without changes in beef demand to exports accounting for 37% of output in the *ALTP60* scenario.

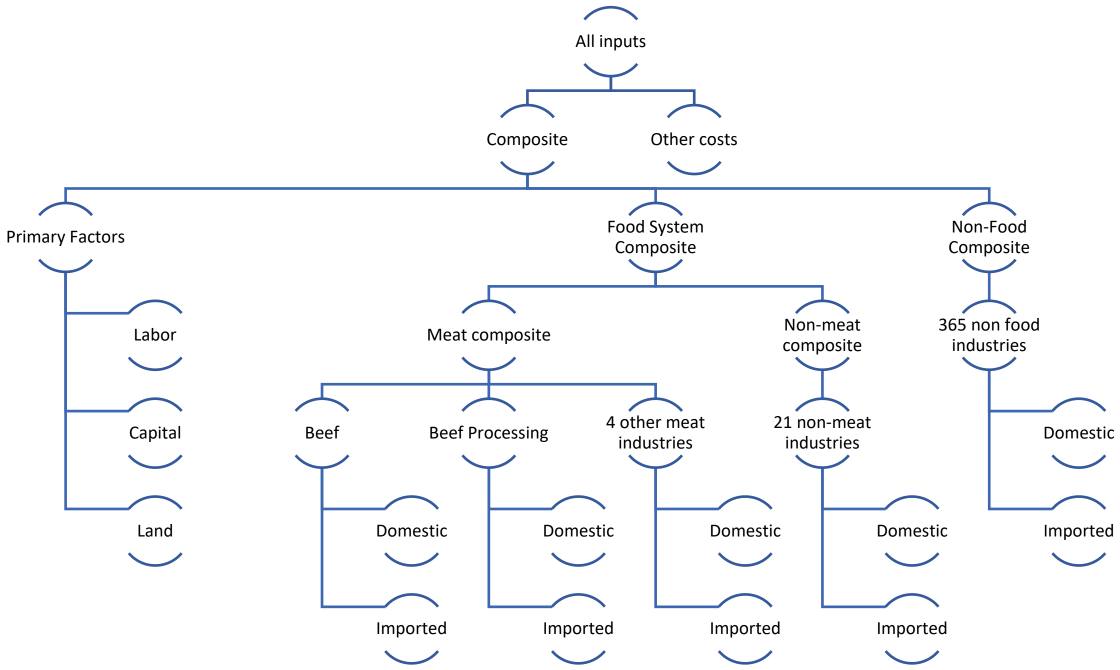

Figure S1 Stylized representation of nesting in USAGE-FOOD production function

CES functions determine substitutability of nodes within each level of the production nesting. Each sector/industry has five levels of nesting, with the lowest level represented by substitutability between domestically produced or imported products.

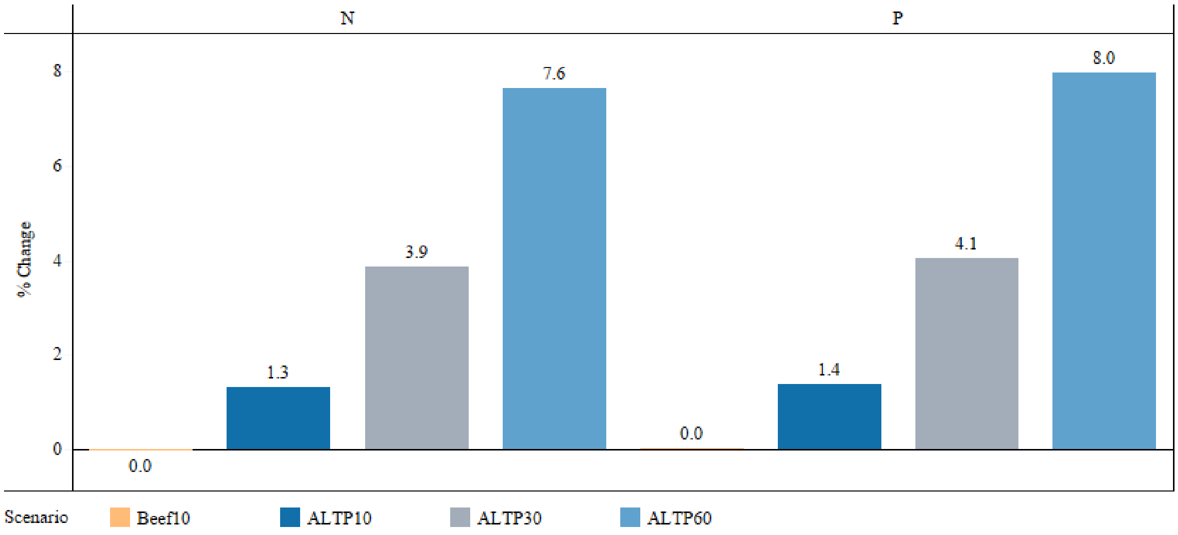

Figure S2 Nitrogen and phosphorous application for U.S. Agriculture by scenario

BEEF10 - 10% reduction in beef expenditure without any novel products
ALTP10-60 - 10-60% of beef expenditure substituted with plant-based alternatives
Demand represents changes in food expenditure in USD; Production represents changes in output in USD. GHG are reported as annual emissions in CO2eq; Water represents changes in blue-water use in km3. Cropland is reported in 1000 ha of harvest area. Blue-water and cropland are not used directly by livestock, but instead for crop production some of which serves as feed.

S3. Model Documentation

Computable General Equilibrium Models

CGE models are quantitative economy-wide models. They describe the interconnected nature of factor markets (e.g., labour, capital, land, etc.), economic activities, and economic outputs. The first CGE models were developed in the 1960s [4], although they were not the first economy-wide models as Input-Output models were developed several decades earlier [5,6]. Input-Output models like CGE models quantified linkages between supply and demand, but they lacked representations of agent behaviour (i.e., households, producers). CGE models expanded on Input-Output models by simulating agent behaviour and motivation, through demand and production functions. CGE models emphasize links provided by competition for scarce resources. CGE models simulate agent behaviour in the following ways:

- Household demand is estimated by assuming households try to maximize their utility of consumption (enjoyment, welfare, etc.) constrained by their budget or income
- Producers are assumed to be cost minimizers subject to constraints on inputs and demand for their products
- Capitalists allocate capital across economic sectors such that they maximize their returns

Modern CGE models further improve on alternative economy-wide models by considering international trade, as well as the role of government in the economy. CGE models incorporate exchange rates, which allows them to simulate an open economy with imports and exports. CGE models also simulate the public-sector, and how taxation and public spending impacts public use of resources as well as the availability and distribution of resources for private use.

Given these different agents behaviour and resource allocation, CGE models then find an equilibrium solution where prices adjust until supply and demand in product and factor markets equalize. CGE models are based on economic theory. They assume profit and utility maximization, and rational behaviour. While the economic concept of utility is broad and encompasses more than just monetary benefits, the results of data driven economic models should be questioned and put into a broader context of social and cultural norms.

What follows are more detailed technical description of the USAGE-Food model, and work undertaken to update USAGE to simulate these scenarios. This technical description is drawn primarily from Mason-D’Croz et al.[7] and the technical appendices in Dixon et al.[8].

General Description of USAGE

USAGE is a detailed CGE model of the U.S. economy, developed over the last 15 years at the Centre of Policy Studies. It has been applied in many studies[9–14], by and on behalf of: the U.S. International Trade Commission; the U.S. Departments of Commerce, Homeland Security, Agriculture, Energy and Transportation; the Canadian Government; and private sector organizations in the U.S. such as the Mitre Corporation and the Cato Institute. Topics have included: terrorism; stimulus policy; trade; immigration; energy; transport infrastructure spending; model validation; model development; and health.

Features that USAGE shares with most other single-country CGE models are that:

- industries and capital creators choose intermediate and primary factor inputs to minimize the costs of supplying any given amounts of output and new capital subject to constant-returns-to-scale production functions with CES nests;
- households choose their consumption bundle to maximize utility subject to factor incomes plus transfers less taxes;
- all agents treat domestic and imported varieties of any commodity as imperfect substitutes [15];

- exporters face downward sloping demand curves for their products;
- commodity and factor prices in each period are determined by the interaction of demand and supply;
- the quantity of capital created for each industry responds to expected rates of return formed using either forward-looking or static specifications; and
- the economy evolves from period to period driven by capital accumulation and exogenously given paths for population, technology, consumer preferences, world trading conditions and policy objectives.

USAGE assesses the effects of a policy by comparing the path of the economy with the policy in place with an explicit baseline path, without the policy. Thus, a simulation of the effects of a policy shock requires two runs of the model: the baseline run, and a policy run. The baseline run is intended to be a plausible forecast. It builds in macro and energy forecasts from the U.S. Energy Information Administration and trends in other variables such as technology and preferences [see, for example Dixon 2017[16]]. Policy runs generate deviations away from the baseline caused by policies under consideration. In the simulations reported in this paper the main elements of the baseline for the period 2020 to 2025 are:

- average annual growth rates in: real GDP of 2.4 percent;
- consumer prices of 2.3 percent;
- employment of 1.1 percent; and
- total factor productivity of 0.9 percent

Policy runs generate deviations away from the baseline caused by policies under consideration. For this project, these policies encapsulate beef-demand-reduction programs. For the most part, we report the effects of policies as percentage deviations from the baseline solution. The deviation results on which we focus are only slightly sensitive to the details of the baseline

There are different versions of USAGE. These versions start from a core model and are modified to facilitate different analyses. For this project, we have created a version of the model that we refer to as USAGE-Food. This version has 392 industries/commodities and are aggregated to facilitate reporting and analysis. The full list of commodities and aggregation are reported in Table S12.

In the current version of USAGE-Food, we allow for limited conversion of cattle-ranching land to land for other agricultural purposes. This smooths out the response of land rental rates to changes in the composition of agricultural output.

In creating USAGE-Food, the core model has been modified by adding Food nests in the household utility function (for more details on the theory see the technical description of the Utility Function). These facilitate the analysis of policies that cause households to shift their demand from one food commodity (Beef products in this project) to other food commodities. To do this it was first necessary to disaggregate the beef value chain from other animal meat value chains (e.g., pork and lamb). Direct purchasers by households account for only about 63 percent of the sales of beef products in the U.S. (excluding intra-industry sales). Another 28 percent occurs via industries such as restaurants, hospitals and colleges that serve food. The remainder goes to industries such as pet food. To allow for changes in the commodity composition of served food, we added food nests to industry production functions. Table S12 lists the commodities in the food nests and the industries that we identified as food servers. Using food nests production and use was split into 6 meat commodities and 21 non-meat food commodities, as summarized in Table S4.

Table S4 Food commodities within food sub-sets in USAGE-Food

Food groupings	Food commodities
**Meat**	PoultryEgg
	FishHuntTrap
	BeefProc
	OthAnimProc
	PoultryProc
	Seafood
**Non-meat**	WetCornMill
	VegMelonFarm
	FruitNutFarm
	FlourMillMalt
	SoyOilseedProc
	FatsOils
	BreakCereal
	SugarConfec
	FrozFood
	FrtVegCanning
	MilkButter
	Cheese
	DryCondEvapDairy
	IceCream
	BreadBakery
	CookiePasta
	SnackFood
	CoffTea
	FlavorSyrup
	SeasDressing
	OthrFoodManu

Updating Food demand in USAGE

To improve the representation of consumer demand in USAGE we reviewed food demand studies, to synthesize, and update consumer demand elasticities in USAGE. This was done to both better capture the relationship of own-prices and food demand, as well as substitution patterns, to improve the way the nesting of food commodities is handled.

We based these updates primarily on five demand studies.[17–21]

1. Orkent and Alston (2010,2011,2012) is our primary source of data and is a food demand study of the USA, by economists at the U.S. Department of Agriculture Economic Research Service. This study included a review of food demand studies of the USA, as well as estimated demand elasticities using different econometric models, as well as different data sources.
2. Green et al. (2013) Is a systematic review with meta-regression of demand studies, and estimates own-price elasticities of food commodities globally, by income-level of countries

3. Cornelsen et al. (2015) is a follow up to Green et al. and estimates cross-price elasticities to understand substitution patterns better.

We compared the elasticities estimated and presented in the various reports by Orkent and Alston to the estimations from Green et al., and Cornelsen et al., and found the estimates by Orkent and Alston to be consistent with the elasticity ranges for high income countries in these two studies. There were a few divergences from the high-income average elasticities, particularly with USA demand for dairy and meat, where consumers appear to be less price sensitive than the high-income country average.

These studies suggest three things:

1. The own-price elasticity of demand for a meat item at a disaggregated level is about -1. This means that if the price of Beef for example rises by 1 percent holding constant the price of other meat commodities, then the demand for beef falls by about 1 percent.
2. The own-price elasticity for meat in general is about -0.6. This means that if the price of beef together with that of all other meat products rises by 1 percent, then the demand for beef falls by only about 0.6 percent.
3. The cross-price elasticities are relatively small and can be of either sign. This means that if the price of poultry rises by 1 percent holding constant the prices of other meat products, then the demand for beef may either rise or fall but the effect is likely to be small.

We introduced these stylized econometric findings to USAGE by adopting appropriate parameter values in a 3-level nested utility function described in more detail below.

Production Functions

In standard versions of USAGE, industry production functions have 3 levels of nests.

1. At the first level, output of an industry is a function of *Composite genuine input* and *Other costs*. *Composite genuine input* consists of primary factors and materials. Using these inputs, uses up resources. *Other costs* are an artificial input used to fill in discrepancies between the total observed cost of inputs and the observed value of output.
2. At the second level, *Composite genuine input* is a function of *Primary-factor input* and inputs of *intermediates undifferentiated* by source.
3. At the third level, *Primary-factor input* is a function of labour, capital and land, and *undifferentiated intermediates* are functions of domestic and imported varieties.

To allow substitution effects in USAGE-Food in industries such as restaurants between different commodity inputs from the food sector, we modify the production functions to allow for 5 levels of nests. The two extra levels allow for substitution within the food product between Meat and Non-Meat, and then within each of Meat and Non-meat substitution between different types of meat (beef, poultry, etc) and substitution between different type of Non-Meat (vegetables, fruit, grains, etc).

Here we start by setting out the general theory of input demand arising from cost-minimization subject to a 5-level nested production in which all nests are CES. Then we consider the nesting structure in USAGE-Food.

Table S5 Production function with 5-level CES nests: general case

$$X0 = CES1\left(\frac{X1(i)}{A1(i)} \quad i=1,...,C1\right) \qquad (L1P)$$

$$X1(i) = CES2\left(\frac{X2(i,f)}{A2(i,f)} \quad f=1,...,C2(i)\right) \text{ for } i = 1, ..., C1 \qquad (L2P)$$

$$X2(i,f) = CES3\left(\frac{X3(i,f,k)}{A3(i,f,k)} \quad k=1,...,C3(i,f)\right) \text{ for } i = 1, ..., C1 \text{ and } f = 1, ..., C2(i) \qquad (L3P)$$

$$X3(i,f,k) = CES4\left(\frac{X4(i,f,k,s)}{A4(i,f,k,s)} \quad s=1,...,C4(i,f,k)\right) \qquad (L4P)$$

for i = 1, ..., C1; f = 1, ..., C2(i); k = 1, ..., C3(i,f)

$$X4(i,f,k,s) = CES5\left(\frac{X5(i,f,k,s,h)}{A5(i,f,k,s,h)} \quad h=1,...,C5(i,f,k,s)\right)$$

for i = 1, ..., C1; f = 1, ..., C2(i); k = 1, ..., C3(i,f), s=1, ...,C4(i,f,k)

(L5P)

Where

X0 is total inputs to production in an industry.

X1(i) is the ith level-1 input that creates total input. C1 is the number of items at level 1.

X2(i,f) is the fth input in the nest at level 2 that creates the i item at level 1. C2(i) is the number of items in the nest at level 2 that create the i item in level 1.

X3(i,f,k) is the kth input in the nest at level 3 that creates the (i,f) item at level 2. C3(i,f) is the number of items in the nest at level 3 that create the (i,f) item in level 2.

X4(i,f,k,s) is the sth input in the nest at level 4 that creates the (i,f,k) item at level 3. C4(i,f,k) is the number of items in the nest at level 4 that create the (i,f,k) item in level 3.

X5(i,f,k,s,h) is the hth input in the nest at level 5 that creates the (i,f,k,s) item at level 4. C5(i,f,k,s) is the number of items in the nest at level 5 that create the (i,f,k,s) item in level 4.

The A's are input-saving or using technical change or taste change variables.

Table S6 Input-demand functions in percentage change form

Under cost-minimizing assumptions, we obtain:

$$x1(i) - a1(i) = x0 - \sigma1\left(p1(i) - \sum_{t\in C1} S1(t)*p1(t)\right) - \sigma1\left(a1(i) - \sum_{t\in C1} S1(t)*a1(t)\right) \qquad (1)$$

$$\text{for } i = 1,...,C1$$

$$p1(i) = \sum_{f\in C2(i)} S2(i,f)*p2(i,f) + \sum_{f\in C2(i)} S2(i,f)*a2(i,f) \quad \text{for } i=1,...,C1 \qquad (2)$$

$$x2(i,f) - a2(i,f) = x1(i) - \sigma2(i)\left(p2(i,f) - \sum_{t\in C2(i)} S2(i,t)*p2(i,t)\right)$$

$$-\sigma2(i)\left(a2(i,f) - \sum_{t\in C2(i)} S2(i,t)*a2(i,t)\right) \qquad (3)$$

$$\text{for } i=1,...,C1; f=1,...,C2(i)$$

$$p2(i,f) = \sum_{k\in C3(i,f)} S3(i,f,k)*p3(i,f,k) + \sum_{k\in C3(i,f)} S3(i,f,k)*a3(i,f,k) \qquad (4)$$

$$\text{for } i=1,...,C1; f=1,...,C2(i)$$

$$x3(i,f,k) - a3(i,f,k) = x2(i,f) - \sigma 3(i,f)\left(p3(i,f,k) - \sum_{t \in C3(i,f)} S3(i,f,t) * p3(i,f,t) \right)$$

$$- \sigma 3(i,f)\left(a3(i,f,k) - \sum_{t \in C3(i,f)} S3(i,f,t) * a3(i,f,t) \right) \quad (5)$$

$$\text{for } i = 1,...,C1; f = 1,...,C2(i); k = 1,...,C3(i,f)$$

$$p3(i,f,k) = \sum_{s \in C4(i,f,k)} S4(i,f,k,s) * p4(i,f,k,s) + \sum_{s \in C4(i,f,k)} S4(i,f,k,s) * a4(i,f,k,s) \quad (6)$$

$$\text{for } i = 1,...,C1; f = 1,...,C2(i); k = 1,...,C3(i,f)$$

$$x4(i,f,k,s) - a4(i,f,k,s) = x3(i,f,k)$$

$$- \sigma 4(i,f,k)\left(p4(i,f,k,s) - \sum_{t \in C4(i,f,k)} S4(i,f,k,t) * p4(i,f,k,t) \right)$$

$$- \sigma 4(i,f,k)\left(a4(i,f,k,s) - \sum_{t \in C4(i,f,k)} S4(i,f,k,t) * a4(i,f,k,t) \right) \quad (7)$$

$$\text{for } i = 1,...,C1; f = 1,...,C2(i); k = 1,...,C3(i,f); s = 1,...,C4(i,f,k)$$

$$p4(i,f,k,s) = \sum_{h \in C5(i,f,k,s)} S5(i,f,k,s,h) * p5(i,f,k,s,h) + \sum_{s \in C5(i,f,k,s)} S5(i,f,k,s,h) * a5(i,f,k,s,h)$$

$$\text{for } i = 1,...,C1; f = 1,...,C2(i); k = 1,...,C3(i,f); s = 1,...,C4(i,f,k)$$

`` (8)

$$x5(i,f,k,s,h) - a5(i,f,k,s,h) = x4(i,f,k,s)$$

$$- \sigma 5(i,f,k,s)\left(p5(i,f,k,s,h) - \sum_{t \in C5(i,f,k,s)} S5(i,f,k,t) * p5(i,f,k,s,t) \right)$$

$$- \sigma 5(i,f,k,s)\left(a5(i,f,k,s,h) - \sum_{t \in C5(i,f,k,s)} S5(i,f,k,t) * a4(i,f,k,s,t) \right)$$

$$\text{for } i = 1,...,C1; f = 1,...,C2(i); k = 1,...,C3(i,f); s = 1,...,C4(i,f,k); h = 1,...,C5(i,f,k,s$$

(9)

In these equations the lowercase x, a and p variables refer to percentage changes in quantities, prices, and technology variables. The uppercase S's refer to cost shares which can be computed from input-output data. S1(i) is the share of level-1 input i in the cost of all level-1 inputs, e.g. the share of Composite genuine input in the total cost of Composite genuine input and Other cost. S2(i,f) is the share in the total cost of level-1 input i accounted for by the fth input in the nest at level 2 that makes up level-1 input i. S3(i,f k) is the share in the total cost of level-2 input (i,f) accounted for by the kth input in the nest at level 3 that makes up level-2 input (i,f). S4(i,f,k,s) is the share in the total cost of level-3 input (i,f,k) accounted for by the sth input in the nest at level 4 that makes up level-3 input (i,f,k). S5(i,f,k,s,h) is the share in the total cost of level-4 input (i,f,k,s) accounted for by the hth input in the nest at level 5 that makes up level-4 input (i,f,k,s). The parameters σ1, σ2(i), σ3(i,f), σ4(i,f,k) and σ5(i,f,k,s) are substitution elasticities occurring in the 5 nests.

Table S7 indicates the nesting structure in the production function for an industry in USAGE-Food, and describes which items appear in each nest. X0 is total input for the industry.

Table S7 Nesting assumptions for an industry production function in USAGE-Food:

(A). Symbolic representation

LEVEL0	LEVEL1	LEVEL2	LEVEL3	LEVEL4	LEVEL5
X0	X1(1)	X2(1,1)	X3(1,1,1)	X4(1,1,1,1)	X5(1,1,1,1,1)
			X3(1,1,2)	X4(1,1,2,1)	X5(1,1,2,1,1)
			X3(1,1,C3(1,1))	X4(1,1,C3(1,1),1)	X5(1,1,C3(1,1),1,1)
		X2(1,2)	X3(1,2,1)	X4(1,2,1,1)	X5(1,2,1,1,1)
					X5(1,2,1,1,2)
				…	…
				X4(1,2,1,C4(1,2,1))	X5(1,2,1,C4(1,2,1))
					X5(1,2,1,C4(1,2,1),2)
			X3(1,2,2)	X4(1,2,2,1)	X5(1,2,2,1,1)
					X5(1,2,2,1,2)
				…	…
				X4(1,2,2,C4(1,2,2))	X5(1,2,2,C4(1,2,2),1)
					X5(1,2,2,C4(1,2,2),2)
		X2(1,3)	X3(1,3,1)	X4(1,3,1,1)	X5(1,3,1,1,1)
					X5(1,3,1,1,2)
		…	…	…	…
		X2(1,C2(1))	X3(1,C2(1),1)	X4(1,C2(1),1,1)	X5(1,C2(1),1,1,1)
					X5(1,C2(1),1,1,2)
	X1(2)	X2(2,1)	X3(2,1,1)	X4(2,1,1,1)	X5(2,1,1,1,1)

(B). Contents of each nest

LEVEL0	LEVEL1	LEVEL2	LEVEL3	LEVEL4	LEVEL5
All input	Composite genuine	Prim Fac	Labour	Labour	Labour
			Capital	Capital	Capital
			land	land	land
		Food	Meat	io com	io com dom
					io com by imp
				…	…
				io com	io com dom
					io com by imp
			Non-meat	io com	io com dom
					io com by imp
				…	…
				io com	io com dom
					io com by imp
		io com	io com	io com	io com dom
					io com by imp
		…	…	…	…
		io com	io com	io com	io com dom
					io com by imp
	Other costs	Other costs	Other costs	Other costs	Other costs

Total input is created by a combination of 2 items at level 1. These two items are Composite genuine input and Other costs, denoted by X1(1) and X1(2). Thus, C1 = 2, see Table S8. This is the number of items at level 1 that go to make up total input.

Table S8 Number of items in the production function nests in USAGE-Food

LEVEL1	LEVEL2	LEVEL3	LEVEL4	LEVEL5
C1=2	C2(1)= 367[(a)]	C3(1,1) = 3[(b)]	C4(1,1,k)=1<br>for k∈ C3(1,1)	C5(1,1,k,1)=1<br>for k∈ C3(1,1)
		C3(1,2) = 2[(b)]	C4(1,2,1)=6[(c)]	C5(1,2,1,k)=2<br>for k ∈ C4(1,2,1)
			C4(1,2,2)=21[(c)]	C5(1,2,2,k)=2<br>for k ∈ C4(1,2,2)
		C3(1,k)=1[(b)]<br>for k = 3, …, C2(1)	C4(1,k,1)=1<br>for k = 3, …, C2(1)	C5(1,k,1,1)=2<br>for all k= 3, … C2(1)
	C2(2)= 1	C3(2,1)=1	C4(2,1,1)= 1	C5(2,1,1,1)=1

(A) USAGE-Food identifies 392 commodities at the input-output level (see Table A2.1). Of these, 27 are food items (see Table 1.1). Thus, there are 366 commodity inputs at level 2, 365 commodities defined at the input-output level plus Food. These commodity inputs feed into the Genuine input X1(1). The composite primary factor input also appears in the level 2 nest that makes the composite Genuine input. Thus, C2(1) = 367.

(B) The 367 items in C2(1) are split into 365 input-output commodities; and 2 FOOD commodities (Meat and Non-meat); and 3 primary factor commodity.

(C) The first FOOD commodity is split into the 6 Meat commodities and the second FOOD commodity is split into the 21 Non-meat food commodities

Because there are two items at level 1 there must be 2 nests at level 2. In USAGE-Food, the first nest at level 2, that is the nest that creates the first item at level 1, contains C2(1) items. These items are: Primary factor, denoted by X2(1,1); Composite food, denoted by X2(1,2); and C2(1) -2 other intermediate inputs, denoted by X2(1,3), ..., X2(1,C2(1)). The second nest at level 2 contains just one item [C2(2) = 1]. This is simply Other costs repeated from level 1 but now denoted by X2(2,1), that is the quantity of the first (and only) input at level 2 that makes up the second input at level 1.

Because there are C2(1)+C2(2) items at level 2 there must be C2(1)+C2(2) nests at level 3 and because C2(2) = 1, Table S7 shows C2(1)+1 nests at level 3. In USAGE-Food, the first nest at level 3, that is the nest that creates the first item [X2(1,1)] at level 2, contains three items [that is C3(1,1) =3]. These items are the constituents of Primary factors, namely labour, capital, and land, denoted by X3(1,1,1), ..., X3(1,1,C3(1,1)). The second nest at level 3, that is the nest that creates the second item [X2(1,2)] at level 2 contains 2 items [C3(1,2) = 2]. The 2 items are sub-categories of Food: Meat and Non-Meat. The next C2(1)-2 nests at level 3 each contain only one item, which is simply a renamed input-output commodity from level 2. The final nest in level 3 contains only one item, which is simply Other costs renamed from level 2 as X3(2,1,1).

Because there are $C3(1,1)+C3(1,2)+\sum_{k=3}^{C2(1)} C3(1,k)+C3(2,1)$ items at level 3 there must be the same number of nests at level 4. The first C3(1,1) of these nests each contain just one item. The item in the first of these nests is Labour renamed as X4(1,1,1,1). The item in the second nest is Capital, renamed as X4(1,1,2,1). The item in the C3(1,1) nest, that is the third nest, is Land, renamed as X4(1,1,C3(1,1),1). In our notation, C4(1,1,k) = 1 for k = 1, …C3(1,1).

The next two nests at level 4 [C3(1,2)=2] comprise input-output food commodities. There are 6 input-output commodities in the first, corresponding to the Meat sub-category of Food and 21 in the second, corresponding to the Non-meat sub-category of Food (see Table S4).

The next C2(1)-2 nests at level 4 [corresponding to $\sum_{k=3}^{C2(1)} C3(1,k)$] each contain only one item, which is simply a renamed item from level 3: X4(1,3,1,1), X4(1,4,1,1), …, X4(1,C2(1),1,1).

The final nest in level 4 contains only one item, which is simply Other costs renamed from level 3 as X4(2,1,1,1).

Because there are $\sum_{k=1}^{C3(1,1)} C4(1,1,k)+C4(1,2,1)+C4(1,2,2)+\sum_{k=3}^{C2(1)} C4(1,k,1)+C4(2,1,1)$ items at level 4 there must be the same number of nests at level 5. The first $\sum_{k=1}^{C3(1,1)} C4(1,1,k)$ of these nests each contains just one primary factor item, renamed from level 4. The next $C4(1,2,1)+C4(1,2,2)$ $+\sum_{k=3}^{C2(1)} C4(1,k,1)$ nests each have two items, the domestic and imported versions of the corresponding input-output commodities identified in level 4. Consider for example, the input-output commodity Flour. The two associated items in level 5 are:

X5("Composite genuine", "Food", "Non-Meat", "flour", "domestic") and

X5("Composite genuine", "Food", "Non-Meat", "flour", "imported").

In our notation, C5(1,2,3,k) = 2 for k = Flour.

Utility Function

In specifying household demands for commodities in USAGE-Food, we use a 4-level utility function: Stone-Geary at the top level with 3 underlying CES nests.

Table S9 4-Level Utility Function

$$U = \sum_i B(i) * \ln\left(\frac{X1(i)}{Q} - G(i)\right) \qquad \text{(L1U)}$$

$$X1(i) = CES2\left(\frac{X2(i,f)}{A2(i,f)} \quad f = 1,...,C2(i)\right) \text{ for } i = 1, ..., C1 \qquad \text{(L2U)}$$

$$X2(i,f) = CES3\left(\frac{X3(i,f,k)}{A3(i,f,k)} \quad k = 1,...,C3(i,f)\right) \text{ for } i = 1, ..., C1 \text{ and } f = 1, ..., C2(i) \qquad \text{(L3U)}$$

$$X3(i,f,k) = CES4\left(\frac{X4(i,f,k,s)}{A4(i,f,k,s)} \quad s = 1,...,C3(i,f,k)\right) \text{ for } i = 1, ..., C1,\ f = 1, ..., C2(i) \text{ and } k = 1, ..., C3(i,f) \qquad \text{(L4U)}$$

where

Q is number of households;
B(i) is the marginal budget share for commodity i;
G(i) is the household per capita subsistence requirement of commodity i;
X1(i) is total household consumption of level-1 commodity i;
X2(i,f) is total household consumption of level-2 commodity i,f, the fth commodity in the nest that generates evel-1 commodity i;
X3(i,f,k) is total household consumption of level-3 commodity i,f,k, the kth commodity in the nest that generates level-2 commodity i,f; and
X4(i,f,k,s) is total household consumption of level-4 commodity i,f,k,s, the sth commodity in the nest that generates level-3 commodity i,f,k; and
the A's are preference-change variables.

Table S10 indicates the nesting structure in USAGE-Food. U is total utility specified by L(1). Total utility is created by a combination of 366 items at level 1. These are per household consumption of 365 non-food input-output commodities denoted by X1(1) to X1(365), and composite food denoted by X1(366). Thus, C1 = 366. This is the number of items at level 1 that go to make up total utility.

Table S10 Nesting assumptions for consumer utility in USAGE-Food

(A). Symbolic representation

LEVEL0	LEVEL1	LEVEL2	LEVEL3	LEVEL4
U	X1(1)	X2(1,1)	X3(1,1,1)	X4(1,1,1,1)<br>X4(1,1,1,2)
	…	…	…	…
	X1(C1(1))	X2(C1(1),1)	X3(C1(1),1,1)	X4(C1(1),1,1,1)<br>X4(C1(1),1,1, 2)
	X1(2)	X2(2,1)	X3(2,1,1)	X4(2,1,1,1)<br>X4(2,1,1,2)
			…	…
			X3(2,1,C3(2,1))	X4(2,1,C3(2,1),1)<br>X4(2,1,C3(2,1),2)
		X2(2,2)	X3(2,2,1)	X4(2,2,1,1)<br>X4(2,2,1,2)
			…	…
			X3(2,2,C3(2,2))	X4(2,2,C3(2,2),1)<br>X4(2,2,C3(2,2),2)

(B). Contents of each nest

LEVEL0	LEVEL1	LEVEL2	LEVEL3	LEVEL4
Utility	io com	io com	io com	io com dom<br>io com imp
	…	…	…	…
	io com	io com	io com	io com dom<br>io com imp
	Food	Flesh	io com	io com dom<br>io com imp
			…	…
			io com	io com dom<br>io com imp
		Non flesh	io com	io com dom<br>io com imp
			…	…
			io com	io com dom<br>io com imp

Because there are 366 items at level 1 there must be 366 nests at level 2. In USAGE-Food, the first 365 nests at level 2, that is the nests that create the first 365 items at level 1, each contain a single item denoted by X2(i,1), i = 1, …, 365. Thus, C2(i) =1 for all i = 1, …, 365. The last nest at level 2 contains the 2 food items: Meat and Non-Meat. Thus C2(366) = 2.

Because there are 367 items at level 2 there must be 367 nests at level 3. In USAGE-Food, the first 365 nests at level 3, that is the nests that create the first 365 items at level 2, each contain a single item denoted by X3(i,1,1), i = 1, …, 365. Thus, C3(i,1) =1 for all i = 1, …, 365. The next nest at level 3 contains the 6 meat items and the last nest contains 21 non-meat items. Thus C3(366,1) = 6 and C3(366,2) = 21.

Because there are 392 items at level 4, Table S11 shows 392 items at level 3. Each of these nests has two items. The two items are the domestic and imported versions of the level-3 input-output commodity.

Table S11 Number of items in the consumer nests in USAGE-Food

LEVEL1	LEVEL2	LEVEL3	LEVEL4
C1=366	C2(k)=1<br>for k = 1, …, 365	C3(k,1)=1<br>for k = 1, …, 365	C4(k,1,1)=2<br>for k = 1, …, 365
	C2(366)=2<br>for k =366	C3(366,1) = 6	C4(366,1.k)=2<br>for k∈ C3(366,1)
		C3(366,2) = 21	C4(366,2,k)=2<br>or k∈ C3(366,2)

Optimization of level-1: cost minimization subject to Stone-Geary utility constraint

For any given level of utility U, households

choose X1(1), …, X1(C1)

to minimize $\sum_i P1(i) * X1(i)$

subject to $U = \sum_i B(i) * \ln\left(\frac{X1(i)}{Q} - G(i)\right)$

First order conditions:

$$P1(i) = \Lambda * B(i) * \frac{1/Q}{X1(i)/Q - G(i)} \tag{10}$$

where Λ is the Lagrangian multiplier.

Rearrange (10) as

$$P1(i) * \left[X1(i)/Q - G(i)\right] = \frac{\Lambda * B(i)}{Q} \tag{11}$$

Sum over i

$$\frac{Y}{Q} - \sum_i P1(i) * G(i) = \frac{\Lambda}{Q} \tag{12}$$

where Y = $\sum_i P1(i) * X1(i)$, that is Y is the household budget.

Substitute (12) into (11). This gives the well known linear expenditure system:

$$\frac{X1(i)}{Q} = G(i) + \frac{B(i)}{P1(i)} * \left[\frac{Y}{Q} - \sum_j P1(j) * G(j)\right] \tag{13}$$

In percentage change form (13) can be written as

$$\frac{X1(i)}{Q}*\left(x1(i)-q\right)=100*dG(i)+\frac{B(i)}{P1(i)}*\frac{Y}{Q}*\left(\beta(i)+y-p1(i)-q\right)$$
$$-\left\{\frac{B(i)}{P1(i)}*\sum_j P1(j)*G(j)\right\}*\left(\beta(i)-p1(i)\right)-\frac{B(i)}{P1(i)}*\left\{\sum_j P1(j)*G(j)*\left[p1(j)\right]+100*\sum_j P1(j)*dG(j)\right\}$$
(14)

where variables denoted by lowercase symbols are percentage changes in variables denoted by the corresponding uppercase symbols. Notice that we use the change form, dG(i), for G(i). This is because G(i) can be of either sign and may move through zero.

After a considerable amount of tedious but elementary algebra we find that (14) can be rewritten as

$$\left(x1(i)-q\right)=\varepsilon(i)*\left(y-q\right)+\sum_j \eta(i,j)*p1(j)$$
$$+100*Q*dG(i)/X1(i)-\varepsilon(i)*100*\sum_j S1(j)*Q*dG(j)/X1(j)-\varepsilon(i)\frac{1}{F}*\beta(i) \quad (15)$$

where $S1(i)=\frac{P1(i)X1(i)}{Y}$ (16)

$$F=\frac{-Y/Q}{Y/Q-\sum_j P1(j)*G(j)} \quad (17)$$

$\varepsilon(i)=\frac{B(i)}{S1(i)}$ and (18)

$$\eta(i,j)=KD(i,j)*\frac{\varepsilon(i)}{F}-\varepsilon(i)*S1(j)*\left(1+\frac{\varepsilon(j)}{F}\right) \quad (19)$$

S1(i) is the share of i in household expenditure.

F is the negative of the reciprocal of the share of supernumerary expenditure in household expenditure. F is known as the Frisch coefficient.

$\eta(i,j)$ is the elasticity of household demand for commodity i with respect to a change in the price of commodity j.

ε(i) is the expenditure elasticity of household demand for commodity i.

Preference variables

Equation (15) contains two preference-change variables for each of the C1 commodities at level 1. These can be written as $Q*dG(j)/X1(j)$ and β(j). In effect, we reduce this to C1 preference changes by connecting $Q*dG(i)/X1(i)$ and β(i) via C1 new variables a1com(i). We do this by writing:

$$\beta(i)=a1com(i)-\sum_k B(k)*a1com(k) \quad (20)$$

and

$$Q * dG(i) / X1(i) = 0.01\left(1+\frac{\varepsilon(i)}{F}\right)*\left[a1com(i)-\sum_{k} S1(k)*a1com(k)\right] \quad (21)$$

If we set a1com(i) at -1, then via (20) and (21) we are imposing a taste change against commodity i of about 1 percent by reducing both the subsistence and supernumerary consumption of i by about 1 percent. Not surprisingly as demonstrated below, under (20) and (21), (15) reduces to

$$\left(x1(i)-q\right)=\varepsilon(i)*\left(y-q\right)+\sum_{j}\eta(i,j)*p1(j)+a1com(i))-\sum_{k} S1(k)*a1com(k) \quad (22)$$

To demonstrate (22), we start by substituting from (20) and (21) into (15) to obtain

$$\begin{aligned}\left(x1(i)-q\right)&=\varepsilon(i)*\left(y-q\right)+\sum_{j}\eta(i,j)*p1(j)\\ &+\left(1+\frac{\varepsilon(i)}{F}\right)*\left[a1com(i)-\sum_{k} S1(k)*a1com(k)\right]\\ &-\varepsilon(i)*\sum_{j} S1(j)*\left(1+\frac{\varepsilon(j)}{F}\right)*\left[a1com(j)-\sum_{k} S1(k)*a1com(k)\right]\\ &-\varepsilon(i)\frac{1}{F}*\left(a1com(i)-\sum_{k} B(k)*a1com(k)\right)\end{aligned} \quad (23)$$

We rewrite (23) with all of the preference expressions broken into individual terms:

$$\begin{aligned}\left(x1(i)-q\right)&=\varepsilon(i)*\left(y-q\right)+\sum_{j}\eta(i,j)*p1(j)\\ &+a1com(i)+\frac{\varepsilon(i)}{F}*a1com(i)-\sum_{k} S1(k)*a1com(k)-\frac{\varepsilon(i)}{F}*\sum_{k} S1(k)*a1com(k)\\ &-\varepsilon(i)*\sum_{j} S1(j)*a1com(j)-\varepsilon(i)*\sum_{j} S1(j)*\frac{\varepsilon(j)}{F}*a1com(j)\\ &-\varepsilon(i)*\sum_{j} S1(j)*\sum_{k} S1(k)*a1com(k)+\varepsilon(i)*\sum_{j} S1(j)*\frac{\varepsilon(j)}{F}*\sum_{k} S1(k)*a1com(k)\\ &-\varepsilon(i)*\frac{1}{F}*a1com(i)+\varepsilon(i)*\frac{1}{F}*\sum_{k} B(k)*a1com(k)\end{aligned} \quad (24)$$

Then we apply three identities: equation (18); sum of shares equal 1; and the share-weighted sum of expenditure elasticities equals 1. This yields (22).

Consumer demand functions in percentage change form

The complete 4-nest household demand system in percentage change form, similar to the GEMPACK specification in USAGE-Food, is:

$$x1(i)-q=\varepsilon(i)*\left(c-q\right)+\sum_{j}\eta(i,j)*p1(j)+a1com(i)-ave\_a1com \quad \text{for } i = 1, 2, \ldots, C1 \quad (25)$$

$$ave\_a1com=\sum_{k} S1(k)*a1com(k)\ , \quad (26)$$

$$p1(j) = \sum_{f \in C2(j)} S2(j,f) * p2(j,f) + \sum_{f \in C2(j)} S2(j,f) * a2(j,f) \quad \text{for } j = 1,...,C1 \tag{27}$$

$$\begin{aligned} x2(i,f) - a2(i,f) = x1(i) - \sigma2(i)\left(p2(i,f) - \sum_{t \in C2(i)} S2(i,t) * p2(i,t) \right) \\ - \sigma2(i)\left(a2(i,f) - \sum_{t \in C2(i)} S2(i,t) * a2(i,t) \right) \\ \text{for } i = 1,...,C1; f = 1,...,C2(i) \end{aligned} \tag{28}$$

$$\begin{aligned} p2(i,f) = \sum_{k \in C3(i,f)} S3(i,f,k) * p3(i,f,k) + \sum_{k \in C3(i,f)} S3(i,f,k) * a3(i,f,k) \\ \text{for } i = 1,...,C1;\ f = 1,...,C2(i); \end{aligned} \tag{29}$$

$$\begin{aligned} x3(i,f,k) - a3(i,f,k) = x2(i,f) - \sigma3(i,f)\left(p3(i,f,k) - \sum_{t \in C3(i,f)} S3(i,f,t) * p3(i,f,t) \right) \\ - \sigma3(i,f)\left(a3(i,f,k) - \sum_{t \in C3(i,f)} S3(i,f,t) * a3(i,f,t) \right) \\ \text{for } i = 1,...,C1; f = 1,...,C2(i); k = 1,...,C3(i,f) \end{aligned} \tag{30}$$

$$\begin{aligned} p3(i,f,k) = \sum_{s \in C4(i,f,k)} S4(i,f,k,s) * p4(i,f,k,s) + \sum_{s \in C4(i,f,k)} S4(i,f,k,s) * a4(i,f,k,s) \\ \text{for } i = 1,...,C1;\ f = 1,...,C2(i);\ k = 1,...,C3(i,f); \end{aligned} \tag{31}$$

$$\begin{aligned} x4(i,f,k,s) - a4(i,f,k,s) = x3(i,f,k) - \sigma4(i,f,k)\left(p4(i,f,k,s) - \sum_{t \in C4(i,f,k)} S4(i,f,k,t) * p4(i,f,k,t) \right) \\ - \sigma4(i,f,k)\left(a4(i,f,k,s) - \sum_{t \in C4(i,f,k)} S4(i,f,k,t) * a4(i,f,k,t) \right) \\ \text{for } i = 1,...,C1; f = 1,...,C2(i); k = 1,...,C3(i,f); s = 1,...,C4(i,f,k) \end{aligned} \tag{32}$$

where the S coefficients are shares derived from household expenditure data in the input-output tables. These are defined by

$$S4(i,f,k,t) = \frac{V(i,f,k,t)}{\sum_{tt \in C3(i,f,k)} V(i,f,k,tt)}$$

$$S3(i,f,k) = \frac{\sum_{t \in C4(i,f,k)} V(i,f,k,t)}{\sum_{kk \in C3(i,f)} \sum_{t \in C4(i,f,k)} V(i,f,kk,t)}$$

$$S2(i,f) = \frac{\sum_{k \in C3(i,f)} \sum_{t \in C4(i,f,k)} V(i,f,k,t)}{\sum_{ff \in C2(i)} \sum_{k \in C3(i,f)} \sum_{t \in C4(i,f,k)} V(i,ff,k,t)}$$

where V(i,f,k,t) is household expenditure on domestic or imported (t) input-output commodity (i,f,k). For example, expenditure on domestic Flour is written as V(Food,Non-

Meat, Flour, domestic), and expenditure on imported cars is written as V(cars, cars, cars, imported).

Table S12 USAGE commodities and industries with mapping to 45 and 9 sector aggregations

COM/IND	NAME	NAICS DESCRIPTIONS	45 SECTOR MAPPING	9 SECTORS MAPPING
**1**	OilSeedFarm	Oilseed farming	OilSeedFarm	Agriculture
**2**	GrainFarm	Grain farming	GrainFarm	Agriculture
**3**	VegMelonFarm	Vegetable and melon farming	VegMelonFarm	Agriculture
**4**	FruitNutFarm	Fruit and tree nut farming	FruitNutFarm	Agriculture
**5**	GreenNursPrd	Greenhouse, nursery, and floriculture production	GreenNursPrd	Agriculture
**6**	OthCropFarm	Other crop farming	OthCropFarm	Agriculture
**7**	CattRancFarm	Beef cattle ranching and farming, including feedlots and dual-purpose ranching and farming	CattRancFarm	Agriculture
**8**	DairCattProd	Dairy cattle and milk production	DairCattProd	Agriculture
**9**	OtherAnimal	Animal production, except cattle and poultry and eggs	OtherAnimal	Agriculture
**10**	PoultryEgg	Poultry and egg production	PoultryEgg	Agriculture
**11**	ForestLog	Forestry and logging	ForestLog	Agriculture
**12**	FishHuntTrap	Fishing, hunting, and trapping	FishHuntTrap	Agriculture
**13**	AggForSupp	Support activities for agriculture and forestry	AggForSupp	Agriculture
**14**	OIlGas	Oil and gas extraction	Mining	Mining
**15**	Coal	Coal mining	Mining	Mining
**16**	GoldOthMetl	Iron, gold, silver, and other metal ore mining	Mining	Mining
**17**	CopNickMine	Copper, nickel, lead, and zinc mining	Mining	Mining
**18**	Stone	Stone mining and quarrying	Mining	Mining
**19**	OtherNonMetl	Other non-metallic mineral mining and quarrying	Mining	Mining
**20**	OilGasDrill	Drilling oil and gas wells	Mining	Mining
**21**	OthMineSupp	Other support activities for mining	Mining	Mining
**22**	PowerGener	Electric power generation, transmission, and distribution	Utilities	Utilities
**23**	NatGasDist	Natural gas distribution	Utilities	Utilities
**24**	WaterSewage	Water, sewage, and other systems	Utilities	Utilities
**25**	NResMainRepa	Non-residential maintenance and repair	Construction	Construction
**26**	ResMaintRepa	Residential maintenance and repair	Construction	Construction
**27**	HeaCareStruc	Health care structures	Construction	Construction
**28**	ManufStruc	Manufacturing structures	Construction	Construction
**29**	PowComStruc	Power and communication structures	Construction	Construction
**30**	EducVocStruc	Educational and vocational structures	Construction	Construction
**31**	HwayStreets	Highways and streets	Construction	Construction
**32**	ComFarmStruc	Commercial structures, including farm structures	Construction	Construction
**33**	OthNResStruc	Other non-residential structures	Construction	Construction
**34**	SFamResStruc	Single-family residential structures	Construction	Construction
**35**	MFamResStruc	Multifamily residential structures	Construction	Construction
**36**	OthResStruc	Other residential structures	Construction	Construction
**37**	SawWoodPres	Sawmills and wood preservation	ManuOther	ManuOther
**38**	EngWoodProd	Veneer, plywood, and engineered wood product manufacturing	ManuOther	ManuOther
**39**	Millwork	Millwork	ManuOther	ManuOther
**40**	OthWoodProd	All other wood product manufacturing	ManuOther	ManuOther
**41**	ClayRefrac	Clay product and refractory manufacturing	ManuOther	ManuOther
**42**	Glass	Glass and glass product manufacturing	ManuOther	ManuOther
**43**	Cement	Cement manufacturing	ManuOther	ManuOther
**44**	ReadyMix	Ready-mix concrete manufacturing	ManuOther	ManuOther
**45**	ConcPipeBric	Concrete pipe, brick, and block manufacturing	ManuOther	ManuOther
**46**	OthConcPrd	Other concrete product manufacturing	ManuOther	ManuOther
**47**	LimeGypsum	Lime and gypsum product manufacturing	ManuOther	ManuOther
**48**	Abrasives	Abrasive product manufacturing	ManuOther	ManuOther

**49**	CutStonePrd	Cut stone and stone product manufacturing	ManuOther	ManuOther
**50**	GrdMinEarth	Ground or treated mineral and earth manufacturing	ManuOther	ManuOther
**51**	MinWool	Mineral wool manufacturing	ManuOther	ManuOther
**52**	MscNonMetMin	Miscellaneous non-metallic mineral products	ManuOther	ManuOther
**53**	IronStlManuf	Iron and steel mills and ferroalloy manufacturing	ManuOther	ManuOther
**54**	PurchStlProd	Steel product manufacturing from purchased steel	ManuOther	ManuOther
**55**	AlRefManuf	Alumina refining and primary aluminium production	ManuOther	ManuOther
**56**	PurchAlProd	Aluminium product manufacturing from purchased aluminium	ManuOther	ManuOther
**57**	CopperSmelt	Primary smelting and refining of copper	ManuOther	ManuOther
**58**	NonferrMetl	Primary smelting and refining of nonferrous metal (except copper and aluminium	ManuOther	ManuOther
**59**	CopperProd	Copper rolling, drawing, extruding, and alloying	ManuOther	ManuOther
**60**	NonferMetlPr	Nonferrous metal (except copper and aluminium) rolling, drawing, extruding, and alloying	ManuOther	ManuOther
**61**	FerrFoundry	Ferrous metal foundries	ManuOther	ManuOther
**62**	NonFerrFound	Nonferrous metal foundries	ManuOther	ManuOther
**63**	OthForgStmp	All other forging, stamping, and sintering	ManuOther	ManuOther
**64**	RollForming	Custom roll forming	ManuOther	ManuOther
**65**	CrwnMtlStamp	Crown and closure manufacturing and metal stamping	ManuOther	ManuOther
**66**	CutHandTool	Cutlery and hand tool manufacturing	ManuOther	ManuOther
**67**	PlateWork	Plate work and fabricated structural product manufacturing	ManuOther	ManuOther
**68**	OrnArchMetal	Ornamental and architectural metal products manufacturing	ManuOther	ManuOther
**69**	Boiler	Power boiler and heat exchanger manufacturing	ManuOther	ManuOther
**70**	MetalTank	Metal tank (heavy gauge) manufacturing	ManuOther	ManuOther
**71**	MetalCntnr	Metal can, box, and other metal container (light gauge) manufacturing	ManuOther	ManuOther
**72**	Hardware	Hardware manufacturing	ManuOther	ManuOther
**73**	SprnWirePrd	Spring and wire product manufacturing	ManuOther	ManuOther
**74**	MachShops	Machine shops	ManuOther	ManuOther
**75**	ScrewNut	Turned product and screw, nut, and bolt manufacturing	ManuOther	ManuOther
**76**	CoatEngrave	Coating, engraving, heat treating and allied activities	ManuOther	ManuOther
**77**	Valves	Valve and fittings other than plumbing	ManuOther	ManuOther
**78**	Plumbing	Plumbing fixture fitting and trim manufacturing	ManuOther	ManuOther
**79**	BallBearng	Ball and roller bearing manufacturing	ManuOther	ManuOther
**80**	Ammunition	Ammunition, arms, ordnance, and accessories manufacturing	ManuOther	ManuOther
**81**	FabPipeFtng	Fabricated pipe and pipe fitting manufacturing	ManuOther	ManuOther
**82**	OthFabMetl	Other fabricated metal manufacturing	ManuOther	ManuOther
**83**	FarmMach	Farm machinery and equipment manufacturing	ManuOther	ManuOther
**84**	LawnEquip	Lawn and garden equipment manufacturing	ManuOther	ManuOther
**85**	ConstMach	Construction machinery manufacturing	ManuOther	ManuOther
**86**	MinOilMach	Mining and oil and gas field machinery manufacturing	ManuOther	ManuOther
**87**	OthInduMach	Other industrial machinery manufacturing	ManuOther	ManuOther
**88**	PlstRbrMach	Plastics and rubber industry machinery manufacturing	ManuOther	ManuOther
**89**	SemicondMach	Semiconductor machinery manufacturing	ManuOther	ManuOther
**90**	VendingMach	Vending, commercial laundry, and other commercial and service industry machinery manufacturing	ManuOther	ManuOther
**91**	OfficeMach	Office machinery manufacturing	ManuOther	ManuOther
**92**	OptInstLens	Optical instrument and lens manufacturing	ManuOther	ManuOther

**93**	PhotoEquip	Photographic and photocopying equipment manufacturing	ManuOther	ManuOther
**94**	AirPurVentil	Air purification and ventilation equipment manufacturing	ManuOther	ManuOther
**95**	HeatingEq	Heating equipment (except warm air furnaces) manufacturing	ManuOther	ManuOther
**96**	ACRefrig	Air conditioning, refrigeration, and warm air heating equipment manufacturing	ManuOther	ManuOther
**97**	MoldMfg	Industrial mould manufacturing	ManuOther	ManuOther
**98**	RollMillMach	Metal cutting and forming machine tool manufacturing	ManuOther	ManuOther
**99**	ToolDieJig	Special tool, die, jig, and fixture manufacturing	ManuOther	ManuOther
**100**	MtlWorkMach	Cutting and machine tool accessory, rolling mill, and other metalworking machinery manufacturing	ManuOther	ManuOther
**101**	Turbine	Turbine and turbine generator set units manufacturing	ManuOther	ManuOther
**102**	GearManuf	Speed changer, industrial high-speed drive, and gear manufacturing	ManuOther	ManuOther
**103**	MechPowTrans	Mechanical power transmission equipment manufacturing	ManuOther	ManuOther
**104**	OthEngEquip	Other engine equipment manufacturing	ManuOther	ManuOther
**105**	Pumps	Pump and pumping equipment manufacturing	ManuOther	ManuOther
**106**	AirGasCmprs	Air and gas compressor manufacturing	ManuOther	ManuOther
**107**	MatlHandl	Material handling equipment manufacturing	ManuOther	ManuOther
**108**	PdrivnHandTl	Power-driven hand tool manufacturing	ManuOther	ManuOther
**109**	Scales	Other general purpose machinery manufacturing	ManuOther	ManuOther
**110**	PackngMach	Packaging machinery manufacturing	ManuOther	ManuOther
**111**	IndFurnace	Industrial process furnace and oven manufacturing	ManuOther	ManuOther
**112**	FluidPower	Fluid power process machinery	ManuOther	ManuOther
**113**	Computers	Electronic computer manufacturing	ManuOther	ManuOther
**114**	CmptrStorage	Computer storage device manufacturing	ManuOther	ManuOther
**115**	CompTermin	Computer terminals and other computer peripheral equipment manufacturing	ManuOther	ManuOther
**116**	Telephone	Telephone apparatus manufacturing	ManuOther	ManuOther
**117**	BroadcastEq	Broadcast and wireless communications equipment	ManuOther	ManuOther
**118**	CommunEquip	Other communications equipment manufacturing	ManuOther	ManuOther
**119**	AudVidEquip	Audio and video equipment manufacturing	ManuOther	ManuOther
**120**	OtElectrnic	Other electronic component manufacturing	ManuOther	ManuOther
**121**	Semicondctr	Semiconductor and related device manufacturing	ManuOther	ManuOther
**122**	PrintCircuit	Printed circuit assembly (electronic assembly) manufacturing	ManuOther	ManuOther
**123**	ElectroMedic	Electromedical and electrotherapeutic apparatus manufacturing	ManuOther	ManuOther
**124**	SearchNavig	Search, detection, and navigation instruments manufacturing	ManuOther	ManuOther
**125**	EnviroContrl	Automatic environmental control manufacturing	ManuOther	ManuOther
**126**	ProcVblInsts	Industrial process variable instruments manufacturing	ManuOther	ManuOther
**127**	FluidMeters	Totalizing fluid meter and counting device manufacturing	ManuOther	ManuOther
**128**	ElecTestInst	Electricity and signal testing instruments manufacturing	ManuOther	ManuOther
**129**	LabInsts	Analytical laboratory instrument manufacturing	ManuOther	ManuOther
**130**	RadiationIns	Irradiation apparatus manufacturing	ManuOther	ManuOther
**131**	WatchClock	Watch, clock, and other measuring and controlling device manufacturing	ManuOther	ManuOther

**132**	MagOptiMedia	Manufacturing and reproducing magnetic and optical media	ManuOther	ManuOther
**133**	Lightbulbs	Electric lamp bulb and part manufacturing	ManuOther	ManuOther
**134**	LightFxtr	Lighting fixture manufacturing	ManuOther	ManuOther
**135**	SmAppliaMf	Small electrical appliance manufacturing	ManuOther	ManuOther
**136**	HshldStove	Household cooking appliance manufacturing	ManuOther	ManuOther
**137**	HshldFridge	Household refrigerator and home freezer manufacturing	ManuOther	ManuOther
**138**	HshldLaundry	Household laundry equipment manufacturing	ManuOther	ManuOther
**139**	OthHshldApp	Other major household appliance manufacturing	ManuOther	ManuOther
**140**	PwrTrnsfrmr	Power, distribution, and specialty transformer manufacturing	ManuOther	ManuOther
**141**	MotorGenratr	Motor and generator manufacturing	ManuOther	ManuOther
**142**	Switchboard	Switchgear and switchboard apparatus manufacturing	ManuOther	ManuOther
**143**	Relays	Relay and industrial control manufacturing	ManuOther	ManuOther
**144**	StorBattery	Storage battery manufacturing	ManuOther	ManuOther
**145**	PrimBatter	Primary battery manufacturing	ManuOther	ManuOther
**146**	ComElecWire	Communication and energy wire and cable manufacturing	ManuOther	ManuOther
**147**	WireDevice	Wiring device manufacturing	ManuOther	ManuOther
**148**	CarbonProds	Carbon and graphite product manufacturing	ManuOther	ManuOther
**149**	MsElEquip	All other miscellaneous electrical equipment and component manufacturing	ManuOther	ManuOther
**150**	Autombile	Automobile manufacturing	ManuOther	ManuOther
**151**	LightTruck	Light truck and utility vehicle manufacturing	ManuOther	ManuOther
**152**	HeavyTruck	Heavy duty truck manufacturing	ManuOther	ManuOther
**153**	VehicleBody	Motor vehicle body manufacturing	ManuOther	ManuOther
**154**	TruckTrailer	Truck trailer manufacturing	ManuOther	ManuOther
**155**	MotorHome	Motor home manufacturing	ManuOther	ManuOther
**156**	TravlTrlr	Travel trailer and camper manufacturing	ManuOther	ManuOther
**157**	GasEngPrts	Motor vehicle gasoline engine and engine parts manufacturing	ManuOther	ManuOther
**158**	ElecEngPrts	Motor vehicle electrical and electronic equipment manufacturing	ManuOther	ManuOther
**159**	SteerBrake	Motor vehicle steering, suspension component (except spring)	ManuOther	ManuOther
**160**	PwrTrainPrts	Motor vehicle transmission and power train parts manufacturing	ManuOther	ManuOther
**161**	SeatingInter	Motor vehicle seating and interior trim manufacturing	ManuOther	ManuOther
**162**	AutoMtlStamp	Motor vehicle metal stamping	ManuOther	ManuOther
**163**	OthAuto	Other motor vehicle parts manufacturing	ManuOther	ManuOther
**164**	Aircraft	Aircraft manufacturing	ManuOther	ManuOther
**165**	AirEngines	Aircraft engine and engine parts manufacturing	ManuOther	ManuOther
**166**	OthAirParts	Other aircraft parts and auxiliary equipment manufacturing	ManuOther	ManuOther
**167**	Missiles	Guided missile and space vehicle manufacturing	ManuOther	ManuOther
**168**	MissilPrts	Propulsion units and parts for space vehicles and guided mis	ManuOther	ManuOther
**169**	RlrdCars	Railroad rolling stock manufacturing	ManuOther	ManuOther
**170**	Ships	Ship building and repairing	ManuOther	ManuOther
**171**	Boats	Boat building	ManuOther	ManuOther
**172**	MotrBikes	Motorcycle, bicycle, and parts manufacturing	ManuOther	ManuOther
**173**	ArmyTanks	Military armoured vehicle, tank, and tank component manufacturing	ManuOther	ManuOther
**174**	OthrTransEq	All other transportation equipment manufacturing	ManuOther	ManuOther
**175**	WoodKitcCabt	Wood kitchen cabinet and countertop manufacturing	ManuOther	ManuOther
**176**	UphlHldFurn	Upholstered household furniture manufacturing	ManuOther	ManuOther
**177**	NonUpHhlFurn	Non-upholstered wood household furniture	ManuOther	ManuOther

		manufacturing		
**178**	OthInsHhFurn	Other household non-upholstered furniture	ManuOther	ManuOther
**179**	InstFurn	Institutional furniture manufacturing	ManuOther	ManuOther
**180**	OfficeFurn	Office furniture and custom architectural woodwork and millwork	ManuOther	ManuOther
**181**	ShcaseShlv	Showcase, partition, shelving, and locker manufacturing	ManuOther	ManuOther
**182**	OthFurn	Other furniture related product manufacturing	ManuOther	ManuOther
**183**	SrgMedInst	Surgical and medical instrument manufacturing	ManuOther	ManuOther
**184**	SurgAppSupp	Surgical appliance and supplies manufacturing	ManuOther	ManuOther
**185**	DentalEquip	Dental equipment and supplies manufacturing	ManuOther	ManuOther
**186**	Ophthalmic	Ophthalmic goods manufacturing	ManuOther	ManuOther
**187**	DentalLab	Dental laboratories	ManuOther	ManuOther
**188**	Jewelry	Jewelry and silverware manufacturing	ManuOther	ManuOther
**189**	SportGoods	Sporting and athletic goods manufacturing	ManuOther	ManuOther
**190**	Toys	Doll, toy, and game manufacturing	ManuOther	ManuOther
**191**	OfficSupply	Office supplies (except paper) manufacturing	ManuOther	ManuOther
**192**	Signs	Sign manufacturing	ManuOther	ManuOther
**193**	AllOthManuf	All other miscellaneous manufacturing	ManuOther	ManuOther
**194**	DogCatFood	Dog and cat food manufacturing	ManuOther	ManuOther
**195**	OthAnFood	Other animal food manufacturing	ManuOther	ManuOther
**196**	FlourMalMill	Flour milling and malt manufacturing	FlourMalMill	FoodManu
**197**	WetCornMill	Wet corn milling	WetCornMill	FoodManu
**198**	SoyOilProc	Soybean and other oilseed processing	SoyOilProc	FoodManu
**199**	FatsOils	Fats and oils refining and blending	FatsOils	FoodManu
**200**	BrkCereal	Breakfast cereal manufacturing	BrkCereal	FoodManu
**201**	SugarConfec	Sugar and confectionery product manufacturing	SugarConfec	FoodManu
**202**	FrozFood	Frozen food manufacturing	FrozFood	FoodManu
**203**	FrtVegCDry	Fruit and vegetable canning, pickling, and drying	FrtVegCDry	FoodManu
**204**	MilkButter	Fluid milk and butter manufacturing	MilkButter	FoodManu
**205**	Cheese	Cheese manufacturing	Cheese	FoodManu
**206**	DCEDairy	Dry, condensed, and evaporated dairy product manufacturing	DCEDairy	FoodManu
**207**	IceCream	Ice cream and frozen dessert manufacturing	IceCream	FoodManu
**208**	AnimalProc	Animal (except poultry) slaughtering, rendering, and process	AnimalProc	FoodManu
**209**	PoultryProc	Poultry processing	PoultryProc	FoodManu
**210**	Seafood	Seafood product preparation and packaging	Seafood	FoodManu
**211**	BreadBakery	Bread and bakery product manufacturing	BreadBakery	FoodManu
**212**	CookiePasta	Cookie, cracker, pasta, and tortilla manufacturing	CookiePasta	FoodManu
**213**	SnackFood	Snack food manufacturing	SnackFood	FoodManu
**214**	CoffTea	Coffee and tea manufacturing	CoffTea	FoodManu
**215**	FlavorSyrup	Flavouring syrup and concentrate manufacturing	FlavorSyrup	FoodManu
**216**	SeasDressing	Seasoning and dressing manufacturing	SeasDressing	FoodManu
**217**	OthrFoodMf	All other food manufacturing	OthrFoodMf	FoodManu
**218**	SoftDrinks	Soft drink and ice manufacturing	SoftDrinks	FoodManu
**219**	Breweries	Breweries	ManuOther	ManuOther
**220**	Wineries	Wineries	ManuOther	ManuOther
**221**	Distilleries	Distilleries	ManuOther	ManuOther
**222**	Tobacco	Tobacco product manufacturing	ManuOther	ManuOther
**223**	FiberYarn	Fibre, yarn, and thread mills	ManuOther	ManuOther
**224**	FabricMills	Fabric mills	ManuOther	ManuOther
**225**	TextFabrCoat	Textile and fabric finishing and fabric coating mills	ManuOther	ManuOther
**226**	Carpet	Carpet and rug mills	ManuOther	ManuOther
**227**	CurtainLinen	Curtain and linen mills	ManuOther	ManuOther
**228**	OthTextMills	Other textile product mills	ManuOther	ManuOther
**229**	ApparelMf	Apparel manufacturing	ManuOther	ManuOther
**230**	LeatherMf	Leather and allied product manufacturing	ManuOther	ManuOther
**231**	PulpMills	Pulp mills	ManuOther	ManuOther
**232**	Paper	Paper mills	ManuOther	ManuOther

**233**	Paperboard	Paperboard mills	ManuOther	ManuOther
**234**	PprContainer	Paperboard container manufacturing	ManuOther	ManuOther
**235**	PprBagTreat	Paper bag and coated and treated paper manufacturing	ManuOther	ManuOther
**236**	Stationry	Stationery product manufacturing	ManuOther	ManuOther
**237**	SanitPpr	Sanitary paper product manufacturing	ManuOther	ManuOther
**238**	OthPprProd	All other converted paper product manufacturing	ManuOther	ManuOther
**239**	Printing	Printing	ManuOther	ManuOther
**240**	SuppPrint	Support activities for printing	ManuOther	ManuOther
**241**	PetrolRefine	Petroleum refineries	ManuOther	ManuOther
**242**	AsphaltPave	Asphalt paving mixture and block manufacturing	ManuOther	ManuOther
**243**	AsphltShngl	Asphalt shingle and coating materials manufacturing	ManuOther	ManuOther
**244**	OthPetroCoal	Other petroleum and coal products manufacturing	ManuOther	ManuOther
**245**	Petrochem	Petrochemical manufacturing	ManuOther	ManuOther
**246**	IndGas	Industrial gas manufacturing	ManuOther	ManuOther
**247**	SynthDye	Synthetic dye and pigment manufacturing	ManuOther	ManuOther
**248**	OthInorgChem	Other basic inorganic chemical manufacturing	ManuOther	ManuOther
**249**	OthOrgChem	Other basic organic chemical manufacturing	ManuOther	ManuOther
**250**	Plastics	Plastics material and resin manufacturing	ManuOther	ManuOther
**251**	SynRubbFiber	Synthetic rubber and artificial and synthetic fibres and fil	ManuOther	ManuOther
**252**	Fertilizer	Fertilizer manufacturing	ManuOther	ManuOther
**253**	Pesticide	Pesticide and other agricultural chemical manufacturing	ManuOther	ManuOther
**254**	MedicBotanic	Medicinal and botanical manufacturing	ManuOther	ManuOther
**255**	Pharma	Pharmaceutical preparation manufacturing	ManuOther	ManuOther
**256**	InVitroDiag	In-vitro diagnostic substance manufacturing	ManuOther	ManuOther
**257**	BiologicProd	Biological product (except diagnostic) manufacturing	ManuOther	ManuOther
**258**	Paint	Paint and coating manufacturing	ManuOther	ManuOther
**259**	Adhesives	Adhesive manufacturing	ManuOther	ManuOther
**260**	Soap	Soap and cleaning compound manufacturing	ManuOther	ManuOther
**261**	ToiletPrep	Toilet preparation manufacturing	ManuOther	ManuOther
**262**	Ink	Printing ink manufacturing	ManuOther	ManuOther
**263**	OthChemical	All other chemical product and preparation manufacturing	ManuOther	ManuOther
**264**	PlstPacking	Plastics packaging materials and unlaminated film and sheet	ManuOther	ManuOther
**265**	PlstPipe	Plastics pipe, pipe fitting, and unlaminated profile shape m	ManuOther	ManuOther
**266**	LamPlstPlate	Laminated plastics plate, sheet (except packaging)	ManuOther	ManuOther
**267**	Polystyrene	Polystyrene foam product manufacturing	ManuOther	ManuOther
**268**	UrethaneFoam	Urethane and other foam product (except polystyrene) manufacturing	ManuOther	ManuOther
**269**	PlstBottle	Plastics bottle manufacturing	ManuOther	ManuOther
**270**	OthPlastic	Other plastics product manufacturing	ManuOther	ManuOther
**271**	Tires	Tire manufacturing	ManuOther	ManuOther
**272**	RbrPlstHose	Rubber and plastics hoses and belting manufacturing	ManuOther	ManuOther
**273**	OthRbrProd	Other rubber product manufacturing	ManuOther	ManuOther
**274**	WholesaleTr	Wholesale trade	OtherServ	OtherServ
**275**	RetailTr	Other retail	OtherServ	OtherServ
**276**	AirTrans	Air transportation, domestic	OtherServ	OtherServ
**277**	RailTrans	Rail transportation	OtherServ	OtherServ
**278**	WaterTrans	Water transportation, domestic	OtherServ	OtherServ
**279**	TruckTrans	Truck transportation	OtherServ	OtherServ
**280**	GrdPassTrans	Transit and ground passenger transportation	OtherServ	OtherServ
**281**	Pipeline	Pipeline transportation	OtherServ	OtherServ
**282**	ScenSuppTran	Scenic and sightseeing transportation and	OtherServ	OtherServ

		support activities		
**283**	Couriers	Couriers and messengers	OtherServ	OtherServ
**284**	Warehousing	Warehousing and storage	OtherServ	OtherServ
**285**	NewspaperPb	Newspaper publishers	OtherServ	OtherServ
**286**	PerdclPub	Periodical Publishers	OtherServ	OtherServ
**287**	BookPub	Book publishers	OtherServ	OtherServ
**288**	DataPub	Directory, mailing list, and other publishers	OtherServ	OtherServ
**289**	SoftwrPub	Software publishers	OtherServ	OtherServ
**290**	MoviesVideo	Motion picture and video industries	OtherServ	OtherServ
**291**	SoundRecord	Sound recording industries	OtherServ	OtherServ
**292**	RadTVBroad	Radio and television broadcasting	OtherServ	OtherServ
**293**	Cable	Cable and other subscription programming	OtherServ	OtherServ
**294**	WiredTelco	Wired telecommunications carriers	OtherServ	OtherServ
**295**	WirelesTelco	Wireless telecommunications carriers (except satellite)	OtherServ	OtherServ
**296**	SatOthTelco	Satellite, telecommunications resellers, and all other telecommunications	OtherServ	OtherServ
**297**	DataHostServ	Data processing, hosting, and related services	OtherServ	OtherServ
**298**	NewsInfoServ	News syndicates, libraries, archives, and all other information	OtherServ	OtherServ
**299**	NetPubSearch	Internet publishing and broadcasting and Web search portals	OtherServ	OtherServ
**300**	MonetDepCred	Monetary authorities and depository credit intermediation	OtherServ	OtherServ
**301**	NonDepCredit	Non-depository credit intermediation and related activities	OtherServ	OtherServ
**302**	SecComBroker	Securities and commodity contracts intermediation and broker	OtherServ	OtherServ
**303**	OthFinance	Other financial investment activities	OtherServ	OtherServ
**304**	InsCarriers	Insurance carriers	OtherServ	OtherServ
**305**	InsBrokers	Insurance agencies, brokerages, and related activities	OtherServ	OtherServ
**306**	FundsTrusts	Funds, trusts, and other financial vehicles	OtherServ	OtherServ
**307**	Housing	Housing	OtherServ	OtherServ
**308**	OthRealEst	Other real estate	OtherServ	OtherServ
**309**	AutoRental	Automotive equipment rental and leasing	OtherServ	OtherServ
**310**	GenrlRentl	Consumer goods and general rental centres	OtherServ	OtherServ
**311**	MachEquRntl	Commercial and industrial machinery and equipment rental and	OtherServ	OtherServ
**312**	AssetLessors	Lessors of nonfinancial intangible assets	OtherServ	OtherServ
**313**	LegalSvces	Legal services	OtherServ	OtherServ
**314**	CustCptrProg	Custom computer programming services	OtherServ	OtherServ
**315**	cptrSysDesgn	Computer systems design services	OtherServ	OtherServ
**316**	OthCptrSvce	Other computer related services, including facilities manage	OtherServ	OtherServ
**317**	Accounting	Accounting, tax preparation, bookkeeping, and payroll services	OtherServ	OtherServ
**318**	ArchEngSvce	Architectural, engineering, and related services	OtherServ	OtherServ
**319**	DesignSvce	Specialized design services	OtherServ	OtherServ
**320**	MgmtCnsltSv	Management consulting services	OtherServ	OtherServ
**321**	EnvCnsltSvc	Environmental and other technical consulting services	OtherServ	OtherServ
**322**	ResDevelSvc	Scientific research and development services	OtherServ	OtherServ
**323**	Advertising	Advertising, public relations, and related services	OtherServ	OtherServ
**324**	MscProfSvces	Marketing research and all other miscellaneous professional,	OtherServ	OtherServ
**325**	PhotoSvce	Photographic services	OtherServ	OtherServ
**326**	VetSvces	Veterinary services	OtherServ	OtherServ
**327**	CompanyMgmt	Management of companies and enterprises	OtherServ	OtherServ
**328**	OffAdmSvces	Office administrative services	OtherServ	OtherServ
**329**	FacilSupSvc	Facilities support services	OtherServ	OtherServ
**330**	EmplSvce	Employment services	OtherServ	OtherServ

**331**	BusnsSupSvc	Business support services	OtherServ	OtherServ
**332**	TravelSvce	Travel arrangement and reservation services	OtherServ	OtherServ
**333**	DetectivSvce	Investigation and security services	OtherServ	OtherServ
**334**	BldgSvce	Services to buildings and dwellings	OtherServ	OtherServ
**335**	OthSuppSvce	Other support services	OtherServ	OtherServ
**336**	WasteMgmt	Waste management and remediation services	OtherServ	OtherServ
**337**	EleSecSchool	Elementary and secondary schools	OtherServ	OtherServ
**338**	Colleges	Junior colleges, colleges, universities, and professional schools	OtherServ	OtherServ
**339**	OtherEducSv	Other educational services	OtherServ	OtherServ
**340**	Physician	Offices of physicians	HealthServ	HealthServ
**341**	Dentists	Offices of dentists	HealthServ	HealthServ
**342**	OthHealth	Offices of other health practitioners	HealthServ	HealthServ
**343**	Outpatient	Outpatient care centres	HealthServ	HealthServ
**344**	MedDiagLab	Medical and diagnostic laboratories	HealthServ	HealthServ
**345**	HomeHlthSvc	Home health care services	HealthServ	HealthServ
**346**	OthAmbul	Other ambulatory health care services	HealthServ	HealthServ
**347**	Hospitals	Hospitals	HealthServ	HealthServ
**348**	NursingHome	Nursing and community care facilities	HealthServ	HealthServ
**349**	MentlHealth	Residential mental retardation, mental health, substance abuse	HealthServ	HealthServ
**350**	IndFamHealth	Individual and family services	HealthServ	HealthServ
**351**	SocialSvce	Community food, housing, and other relief services	OtherServ	OtherServ
**352**	ChildCare	Child day care services	OtherServ	OtherServ
**353**	PerfArts	Performing arts companies	OtherServ	OtherServ
**354**	SpectSports	Spectator sports	OtherServ	OtherServ
**355**	Promoters	Promoters of performing arts and sports and agents for publishers	OtherServ	OtherServ
**356**	IndArtists	Independent artists, writers, and performers	OtherServ	OtherServ
**357**	MuseumZoo	Museums, historical sites, zoos, and parks	OtherServ	OtherServ
**358**	AmusePark	Amusement parks and arcades	OtherServ	OtherServ
**359**	Gambling	Gambling industries (except casino hotels)	OtherServ	OtherServ
**360**	OthAmuse	Other amusement and recreation industries	OtherServ	OtherServ
**361**	AccHotels	Accommodation	AccHotels	FoodServ
**362**	FullResto	Full-service restaurants	FullResto	FoodServ
**363**	LimResto	Limited-service restaurants	LimResto	FoodServ
**364**	OthFoodDrink	All other food and drinking places	OtherServ	OtherServ
**365**	AutoRepair	Automotive repair and maintenance	OtherServ	OtherServ
**366**	ElEquiRepair	Electronic and precision equipment repair and maintenance	OtherServ	OtherServ
**367**	MachinerRp	Commercial and industrial machinery and equipment repair and	OtherServ	OtherServ
**368**	HhGoodsRpr	Personal and household goods repair and maintenance	OtherServ	OtherServ
**369**	PersCareSvce	Personal care services	OtherServ	OtherServ
**370**	DeathCareSv	Death care services	OtherServ	OtherServ
**371**	CleanLaundry	Dry-cleaning and laundry services	OtherServ	OtherServ
**372**	OthPerSvce	Other personal services	OtherServ	OtherServ
**373**	ReligiousOrg	Religious organizations	OtherServ	OtherServ
**374**	GrantOrg	Grant making, giving, and social advocacy organizations	OtherServ	OtherServ
**375**	CivSocialOr	Civic, social, professional organizations	OtherServ	OtherServ
**376**	PrivHhlds	Private households	OtherServ	OtherServ
**377**	FedGovDef	Federal general government (defence)	OtherServ	OtherServ
**378**	FedGovNonDef	Federal general government (nondefense)	OtherServ	OtherServ
**379**	PostalSvc	Postal service	OtherServ	OtherServ
**380**	OthFedGEnt	Other federal government enterprises	OtherServ	OtherServ
**381**	SLG	State and local general government	OtherServ	OtherServ
**382**	OthSLGEnt	Other state and local government enterprises	OtherServ	OtherServ
**383**	Scrap	Scrap	OtherServ	OtherServ
**384**	Used2HndGds	Used and second-hand goods	OtherServ	OtherServ
**385**	NoncompImprt	Noncomparable imports	OtherServ	OtherServ

**386**	Holiday	Vacation	OtherServ	OtherServ
**387**	FgnHol	Foreign vacation	OtherServ	OtherServ
**388**	ExpTour	Export tourism	OtherServ	OtherServ
**389**	ExpEdu	Export education	OtherServ	OtherServ
**390**	OthNonRes	Other non-residential spending	OtherServ	OtherServ
**391**	AirInt	International air transport	OtherServ	OtherServ
**392**	WatInt	International water transport	OtherServ	OtherServ

S4. Sensitivity of modelling results to changes in export specification of the beef sector

As shown in the main text, particularly Figure 2, reductions in household consumption of beef (through both direct purchases and food-serving establishments) reduce U.S. output of Live cattle and Beef processing. However, the percentage reductions in outputs are less than the percentage reductions in consumption. For example, in the **BEEF10** and **ALTP10** simulations, the consumption reductions are 10 percent, but the output reductions are between 6.9 and 7.6 percent. Output in the beef sector in our simulations is cushioned by sales to pet-food manufacturers and to exports.

We assume that pet-food sales would not be markedly affected by reductions in human consumption of beef products in the U.S. On the other hand, we would expect export sales to react. This is because reductions in U.S. demand for beef lowers the prices of both Live cattle and Beef processing. Lower prices would stimulate export sales.

The simulated extent of the export stimulation depends on export-demand elasticities (sensitivity of foreign demands to changes in prices). In standard simulations, including those in the main text, the export-demand elasticities for Live cattle and Beef processing are set at -3. This means that a one percent reduction in prices stimulates export demand by three percent. There is considerable uncertainty about the values of export-demand elasticities. Thus, it is possible that our standard simulations over- or under-estimate the extent to which the U.S. beef sector could escape the negative effects of domestic demand reduction via increased export sales.

To check whether alternative values for export-demand elasticities lead to significantly different simulation results, we reran the **BEEF10** and **ALTP10** simulations with the export-demand elasticities for Live cattle and Beef processing set at -2, instead of -3. For **BEEF10**, the sensitivity of our results to this variation can be assessed by comparing the second and first columns of results (marked **Sensitivity 1** and **Standard**) in Table S13. Similarly, for **ALTP10** the sensitivity can be assessed by comparing the fifth and fourth columns (again marked **Sensitivity 1** and **Standard**).

As expected, lower export-demand elasticities in **BEEF10** and **ALTP10** lead to simulation results implying greater damage to output, employment and exports for Live cattle and Beef processing. However, the differences in results are very small indicating that our main conclusions are robust with respect to changes in these export-demand elasticities.

In a second pair of sensitivity simulations, we added to the **Sensitivity-1** simulations 10 percent leftward movements in foreign-demand curves for U.S. Live cattle and Beef processing. This means that at any given U.S. price, exports are 10 percent lower. The idea we are capturing is that the preference shift by U.S. households against beef consumption might be duplicated in the rest of the world. The sensitivity of our **BEFF10** results to this world-wide reduction in beef demand can be assessed by comparing the third and second columns of results (marked **Sensitivity 2** and **Sensitivity 1**) in Table S13. For **ALTP10**, the sensitivity can be assessed by comparing the sixth and fifth columns (again marked **Sensitivity 2** and **Sensitivity 1**).

With a reduction in world consumption paralleling the reduction in U.S. consumption, the damage to output and employment in the U.S. beef sector is accentuated. Although exports of Live cattle and Beef processing are about 10 percent lower[1] in the **Sensitivity-2** simulations than in the **Sensitivity-1** simulations, output and employment in these industries are only about 1 percent lower. This reflects low export shares in the sales of U.S. Live cattle and Beef processing. More generally, low connection

[1] Why not exactly 10 percent lower? The supply curves for exports of Live cattle and Beef processing are not flat and, in any case, they shift in response to various minor induced changes in the prices of inputs.

of the beef sector to export markets means that our simulations results are not strongly dependent on our assumptions concerning this sector's export possibilities.

Table S13 Sensitivity of results to changes in the export specification for the beef sector: percentage effects of a 10 percent reduction in household demand for Beef processing products

	BEEF10			ALTP10		
	Standard[a]	Sensitivity 1[b]	Sensitivity 2[c]	Standard[a]	Sensitivity 1[b]	Sensitivity 2[c]
**A) Economy-wide Results**						
GDP	0.0009	0.0007	-0.0004	0.0017	0.0016	0.0004
Household expenditure	-0.0001	-0.0004	-0.0035	0.0002	-0.0002	-0.0032
Aggregate employment	0.0000	0.0000	0.0000	0.0000	0.0000	0.0000
Average wages	-0.0017	-0.0021	-0.0071	-0.0056	-0.0060	-0.0111
**B) Aggregate Food System Results**						
Output						
Agriculture	-0.62	-0.63	-0.79	-0.55	-0.56	-0.71
Food manufacturing	-0.26	-0.27	-0.42	-0.28	-0.29	-0.45
Food services	0.00	0.00	0.00	0.00	0.00	0.01
Employment						
Agriculture	-0.52	-0.54	-0.69	-0.69	-0.70	-0.85
Food manufacturing	-0.19	-0.21	-0.36	-0.55	-0.56	-0.72
Food services	0.00	0.00	0.00	0.00	0.01	0.01
**C) Changes within the Food System**						
Output, Beef Sector						
Live cattle	-6.86	-6.94	-7.98	-6.99	-7.07	-8.07
Beef processing	-7.57	-7.66	-8.73	-7.64	-7.73	-8.76
Employment, Beef Sector						
Live cattle	-7.45	-7.55	-8.66	-7.58	-7.66	-8.74
Beef processing	-7.97	-8.07	-9.20	-8.06	-8.15	-9.24
Exports, Beef Sector						
Live cattle	2.46	1.66	-8.20	2.46	1.66	-8.21
Beef processing	2.51	1.69	-8.18	2.52	1.70	-8.19
Output, Other Meat						
Live poultry	1.00	1.00	0.99	0.19	0.19	0.18
Poultry processing	1.01	1.01	0.99	0.23	0.23	0.21
Other live animals	0.66	0.66	0.67	0.28	0.29	0.29
Other meat processing	1.20	1.20	1.21	0.50	0.50	0.51
Employment, Other Meat						
Live poultry	1.11	1.11	1.10	0.24	0.23	0.22
Poultry processing	1.02	1.02	1.00	0.20	0.19	0.17
Other live animals	0.73	0.73	0.74	0.32	0.32	0.33
Other meat processing	1.27	1.27	1.29	0.53	0.54	0.54
Output, Selected Plant Products						
Grain farms	-0.06	-0.07	-0.12	1.48	1.48	1.44
Flour mill	0.89	0.89	0.91	7.43	7.43	7.49
Maize processing	0.55	0.55	0.57	2.80	2.80	2.83
Oilseed farms	0.25	0.25	0.29	1.86	1.86	1.91
Soya oil processing	0.50	0.50	0.50	3.63	3.64	3.66
Employment, Selected Plant Products						
Grain farms	-0.08	-0.09	-0.14	1.73	1.72	1.68
Flour mill	0.95	0.95	0.97	7.48	7.49	7.54
Maize processing	0.54	0.54	0.56	2.69	2.70	2.73
Oilseed farms	0.28	0.28	0.32	2.31	2.31	2.36
Soya oil processing	0.54	0.54	0.55	4.43	4.43	4.46

[a] Standard. Simulation conducted with standard parameter values, as in Figure 2 in the main text.

[b] Sensitivity 1. Export demand elasticities for Live cattle and Beef processing set at -2, rather than standard values of -3.

[c] Sensitivity 2. Same as sensitivity 1 plus 10% contraction in world demand for U.S. Live cattle and Beef processing.

References

1. Fraser, D. *Understanding animal welfare: the science in its cultural context*. (Wiley-Blackwell, 2008).

2. Doggett, T. Moral Vegetarianism. in *Stanford Encyclopedia of Philosophy* (ed. Zalta, E. N.) (Metaphysics Research Lab, Stanford University, 2018).

3. Fischer, B. *The Ethics of Eating Animals. Usually bad, sometimes wrong, often permissible.* (Routledge, 2020).

4. Johansen, L. *A multi-sectoral study of economic growth*. vol. 82 (North-Holland, 1960).

5. Leontief, W. W. Quantitative Input and Output Relations in the Economic Systems of the United States. *The Review of Economics and Statistics* **18**, 105 (1936).

6. Leontief, W. W. *The structure of American economy, 1919-1929: an empirical application of equilibrium analysis*. (Harvard University Press, 1941).

7. Mason-D'Croz, D. *et al. Pathways to sustainable beef production and demand in the United States*. (2020).

8. Dixon, P. B., Rimmer, M. T. & Mason-D'Croz, D. *Computable General Equilibrium Simulations of the Effects on the U.S. Economy of Reductions in Beef Consumption*. (2020).

9. Dixon, P. B. & Rimmer, M. T. Validating a Detailed, Dynamic CGE Model of the USA. *Economic Record* **86**, 22–34 (2010).

10. Bird, R., Menzies, G., Dixon, P. B. & Rimmer, M. T. The economic costs of US stock mispricing. *Journal of Policy Modeling* **33**, 552–567 (2011).

11. Dixon, P. B., Rimmer, M. T. & Waschik, R. Linking CGE and specialist models: Deriving the implications of highway policy using USAGE-Hwy. *Economic Modelling* **66**, 1–18 (2017).

12. Banse, M. *et al. Factor Markets in General Computable Equilibrium Models*. (2013).

13. Dixon, P. B., Rimmer, M. T. & Waschik, R. G. Evaluating the effects of local content measures in a CGE model: Eliminating the US Buy America(n) programs. *Economic Modelling* **68**, 155–166 (2018).

14. Dixon, P. B., Johnson, M. & Rimmer, M. T. Economy-wide Effects of Reducing Illegal Immigrants in U.S. Employment. *Contemporary Economic Policy* **29**, 14–30 (2011).

15. Armington, P. S. A Theory of Demand for Products Distinguished by Place of Production. *Staff Papers - International Monetary Fund* **16**, 159 (1969).

16. Dixon, P. B., Rimmer, M. T. & Waschik, R. Linking CGE and specialist models: Deriving the implications of highway policy using USAGE-Hwy. *Economic Modelling* **66**, 1–18 (2017).

17. Okrent, A. & Alston, J. M. *Demand for Food in the United States. A review of literature, evaluation of previous estimates and presentation of new estimates of demand*. (2010).

18. Okrent, A. M. & Alston, J. M. *Demand for Food in the United States. A Review of Literature, Evaluation of Previous Estimates, and Presentation of New Estimates of Demand*. (University of California, 2011).

19. Okrent, A. M. & Alston, J. M. *The Demand for Disaggregated Food-Away-from-Home and Food-at-Home Products in the United States*. (2012).

20. Green, R. *et al.* The effect of rising food prices on food consumption: systematic review with meta-regression. *BMJ (Clinical research ed.)* **346**, 1–9 (2013).

21. Cornelsen, L. *et al.* What Happens to Patterns of Food Consumption when Food Prices Change? Evidence from A Systematic Review and Meta-Analysis of Food Price Elasticities Globally. *Health Economics* **24**, 1548–1559 (2015).
