# Supplementary material for: Ethical and economic implications of the adoption of novel plant-based beef substitutes in the USA: a general equilibrium modelling study
Source: Lancet Planet Health. 2022 Aug 3;6(8):e658–69. doi: 10.1016/S2542-5196(22)00169-3 (PMC9364141; doi:10.1016/S2542-5196(22)00169-3)
Supplement: Supplementary appendix [file mmc2.pdf]

THE LANCET
Planetary Health

Supplementary appendix 2

This appendix formed part of the original submission and has been peer reviewed. We post it as supplied by the authors.

Supplement to: Mason-D'Croz D, Barnhill A, Bernstein J, et al. Ethical and economic implications of the adoption of novel plant-based beef substitutes in the USA: a general equilibrium modelling study. *Lancet Planet Health* 2022; **6:** e658–69.
